# Visualizing Plant Responses: Novel Insights Possible Through Affordable Imaging Techniques in the Greenhouse

**DOI:** 10.3390/s24206676

**Published:** 2024-10-17

**Authors:** Matthew M. Conley, Reagan W. Hejl, Desalegn D. Serba, Clinton F. Williams

**Affiliations:** U.S. Arid-Land Agricultural Research Center, U.S. Department of Agriculture, Agricultural Research Service, Maricopa, AZ 85138, USA; reagan.hejl@usda.gov (R.W.H.); des.serba@usda.gov (D.D.S.); clinton.williams@usda.gov (C.F.W.)

**Keywords:** turfgrass research, plant phenotyping, imagery, consumer camera, cover quantification, color analysis, image processing, image corrections, agricultural innovation

## Abstract

Efficient and affordable plant phenotyping methods are an essential response to global climatic pressures. This study demonstrates the continued potential of consumer-grade photography to capture plant phenotypic traits in turfgrass and derive new calculations. Yet the effects of image corrections on individual calculations are often unreported. Turfgrass lysimeters were photographed over 8 weeks using a custom lightbox and consumer-grade camera. Subsequent imagery was analyzed for area of cover, color metrics, and sensitivity to image corrections. Findings were compared to active spectral reflectance data and previously reported measurements of visual quality, productivity, and water use. Results confirm that Red–Green–Blue imagery effectively measures plant treatment effects. Notable correlations were observed for corrected imagery, including between yellow fractional area with human visual quality ratings (r = −0.89), dark green color index with clipping productivity (r = 0.61), and an index combination term with water use (r = −0.60). The calculation of green fractional area correlated with Normalized Difference Vegetation Index (r = 0.91), and its RED reflectance spectra (r = −0.87). A new chromatic ratio correlated with Normalized Difference Red-Edge index (r = 0.90) and its Red-Edge reflectance spectra (r = −0.74), while a new calculation correlated strongest to Near-Infrared (r = 0.90). Additionally, the combined index term significantly differentiated between the treatment effects of date, mowing height, deficit irrigation, and their interactions (*p* < 0.001). Sensitivity and statistical analyses of typical image file formats and corrections that included JPEG, TIFF, geometric lens distortion correction, and color correction were conducted. Findings highlight the need for more standardization in image corrections and to determine the biological relevance of the new image data calculations.

## 1. Introduction

Evidence suggests that human–plant interactions play a crucial role in promoting both physiological well-being and environmental aesthetics, with implications for public health and urban planning [1,2]. Within this context, people primarily gain awareness of the environment through visual perception [3], and the photographic camera is an instrument that can be considered to extend this fundamental human sense [4,5,6]. Modern cameras have evolved to offer robust optical detection, available in consumer-grade products such as the GoPro Hero 12 (GoPro Corporation, SanMateo, CA, USA) or Sony Alpha 9 (Sony Corporation New York, NY, USA), among many others. This benefit is largely attributed to the considerable and sustained investments in academic and commercial optical research which drive advancement for camera products at leading manufacturers [7] such as Canon (Canon Corporation, Melville, NY, USA) and Nikon (Nikon Corporation, Melville, NY, USA). As a result, optoelectronic camera technology provides one of the most sophisticated consumer-available sensor products [8,9,10,11].

Modern digital cameras efficiently record spectral reflectance data as two-dimensional perspective maps that accurately represent real-world objects. The subjects of photography may encompass vegetation, where Red–Green–Blue color (RGB) foliar reflectance is linked to both subjective visual quality and the underlying productive photosystem pigmentation [12,13,14]. Therefore, optical cameras can be expected to provide wide utility in the measurement of plant canopy status and change. They can detect characteristics such as the area of green biomass cover and can resolve subtle color differences presented by plant tissues [15,16,17]. However, the acquisition of scientific data is more widely utilized [18,19,20,21] when it is not only of adequate descriptive quality but also inexpensive and easy to produce, with understandable controls that involve generic parts.

Cameras have been utilized in a wide range of applications across various fields, from industrial fabrication to diverse agricultural and environmental assessments. In industry they have been employed to measure parts [22]. In agriculture and environmental science cameras have been utilized to assess soil moisture [23] and for plant phenotyping [24,25,26,27,28]. They have been instrumental in measuring plant diseases [29], predation effects [30], senescence [31], salt tolerance [32], and drought response [33]. In crop management cameras have been used to determine percent green cover [34,35,36,37,38], make fertilizer recommendations [39], and measure grass-legume overyield [40]. Additionally, cameras have been applied in fruit analysis [41], and in genetic studies to link phenotype to genotype [42,43,44,45]. Most researchers in the turfgrass community follow the work of Richardson and Karcher [35] and use low-cost image methods. In contrast, premier plant phenotyping research often employs expensive sensing equipment (https://terraref.org/, accessed on 10 April 2024), complex experimental facilities (https://www.plantphenomics.org.au/, accessed on 10 April 2024), and robust data corrections that utilize specialized software and advanced mathematics [46,47,48,49]. Yet further improvement in the interpretation of multi-environment data with biological understanding will empower the scaling of plant phenotyping across its many applications [50]. This paper contributes to phenotyping methods by offering straightforward exposure control guidance for consumer camera imagery and enhancing the understanding of image correction efficacy and standardization.

Imagery used for plant phenotyping is a common practice because it can increase data precision, speed, and consistency over traditional manual plant assessments [51]. Additionally, it can offer increased spatial and statistical power over single electronic point measurement collections [52,53]. Leaders in high-throughput plant phenotyping frequently employ advanced engineering and sensing technologies, generating complex datasets that necessitate sophisticated processing, correction, and analysis techniques. [54,55]. This approach is effective in addressing research questions, but it usually has a limited scope of application and may necessitate a team of experts or expensive equipment acquisitions [56,57,58,59]. High-throughput plant phenotyping can be complemented by employing a simplified approach utilizing consumer-grade cameras with basic controls. The inherent complexity of current consumer camera technology offers a promising opportunity for data generation. This approach can effectively address research questions, albeit with some trade-offs, because it often operates on a smaller scale and with reduced throughput. However, it also comes with reduced costs and lower technical requirements, potentially making it useful across many applications. The modality of uncomplicated image-based proximal phenotyping is most suitable for students, commercial managers, or professional researchers conducting experiments of limited size. This use of modern cameras with simple controls enables detection of plant cover area and color qualities, and although outside the scope of this paper, it opens opportunity for further spatial analysis using shape and texture features.

Grasses comprise a significant component of the Earth’s flora [60,61,62], and turfgrasses provide the primary vegetative cover in many places where humans interact [63,64]. They cover approximately two percent of the continental United States [65], or, for instance, thirty percent of metropolitan Albuquerque, New Mexico [66]. The assessment of turfgrass canopy is an essential aspect of the relationship between humans and plants for effectively gaining ecosystem services through sustainable resource utilization.

The visual quality rating of turfgrass is a traditional procedure that follows standardized guidelines (https://www.ntep.org/, accessed on 9 April 2024 [67]). Human visual quality ratings of turfgrass (VQ) as a practice [68] are useful because they include broad subjective information apparent to the complexity of human sensory perception and the cognitive expertise of the expert, although a constraint for a single expert observer to increase accuracy and minimize data variance persists. Separately, NDVI is a well-regarded remotely sensed normalized difference ratio measure of the Red (RED) and Near-Infrared (NIR) spectral reflectance which detects presence of green biomass [69,70,71,72,73,74]. NDVI has been successful in many applications to resolve plant cover [75,76,77], plant nitrogen status [78], plant vigor [79,80], and other attributes of vegetation, including chlorophyll content [81,82], influence of drip irrigation [83] and deficit irrigation [84]. Consequently, VQ and NDVI are both typical assessors of turfgrass that can be used in different environmental conditions and management scenarios [85,86].

This paper builds on related work that shows how imaging is useful in turfgrass research to detect the area of green cover and change in plant establishment over time [87]. This is often achieved by counting green classified pixels and calculating a percentage of green presence [88,89,90,91]. The process is enabled by computers and can be partially automated using programming scripts or recorded macros [35]. Richardson and Karcher [92,93], and Zhang [94] have presented the common approach of using a lightbox for imaging turfgrass, while recent results show how low-cost imaging outside the lightbox can resolve genetic performance in the greenhouse [95]. Once data are digitized in silico, many additional and more advanced opportunities for processing and analysis become available [49,96,97,98,99,100].

To enable the accurate interpretation of visual information, a primary prerequisite is establishing relationships between image-based data and reliable biological metrics. Vegetation metrics calculated from a single set of image data are automatically dependent to some extent, and without a reference they do not provide a measure of alignment with the underlying biological phenomena. Therefore, the isolated analysis of an individual plant image collection may not account for biases induced by the camera, photographic exposure, environmental variations, or computational process. However, once the verification of a specific camera profile response to the plant phenotype trait is obtained, subsequent image data may hold increased independent value and no longer require related attribution. This idea extends to the image correction process implemented post-collection and includes the image file format. Understanding how a specific lossy file compression like JPEG (Joint Photographic Experts Group), image geometric lens distortion correction (LC), and color correction (CC) may affect phenotypic detection ability relative to various image-based calculations ensures experimental control and improves discovery potential. Our literature review did not reveal studies that directly examined the effects of image corrections on image-based calculations in relation to turfgrass phenotyping metrics.

This research aims to validate the efficacy of modern consumer-grade cameras, when used with basic image controls, for plant phenotyping applications. We affirm such imagery can provide data representative of conventional phenotyping metrics, such as human-assessed visual quality (VQ), and we investigate the representation of biomass productivity, water use, and independently measured multi-spectral reflectance using expert-assessed turfgrass VQ and NDVI as benchmarks [101,102,103,104]. Additionally, we example the potential for deriving novel relationships from image-based metrics. By comparing camera-derived information with established metrics, this study seeks to demonstrate the viability of accessible imaging technology for quantitative plant assessment. Furthermore, we evaluate the impact of two typical image file formats and two common corrections on the results.

This work seeks to advance current image analysis methods and provide practical guidance for image-based plant phenotyping. It presents two new image calculations, demonstrates the importance of yellow fraction classification, and illustrates correction effects for two image formats and two corrections derived from reproducible, cost-free software.

Main novelty and contribution list:

- Guidance is given for simple image-based phenotyping measurements;
- Two new image metrics, HSVi and BA ratio are presented;
- The importance of a yellow color classification is demonstrated;
- Analysis of image corrections highlights need for standardization.

## 2. Materials and Methods

An economical (less than USD 1000) Nikon N1 aw1 digital single-lens reflex (DSLR) action camera with 11–27.5 mm f 3.5–5.6 NIKKOR lens, and custom-made LED lightbox were used to investigate 72 custom PVC lysimeters (15.2 cm diameter × 30.5 cm depth) growing ‘TifTuf’ bermudagrass (*Cynodon dactylon* × *C. transvaalensis* Burt Davy). The turfgrass was grown concurrently at the U.S. Arid-Land Agricultural Research Center in Maricopa, AZ, USA, as 2 sets of 36 lysimeters inside 2 greenhouses during the Autumn season of 2023. Additional management information is in Appendix A. The lysimeters were treated with four mowing heights (2.5, 5.0, 7.5, and 10.0 cm) and three water application levels [(100 × ET_a_ (actual evapotranspiration), 65 × ET_a_, and 30 × ET_a_)], and evaluated for visual quality, clipping productivity (biomass), and water use of the 100 × ET_a_ treatment (gravimetric loss). The experiment is described in detail in Hejl et al. [105]. Appendix A contains descriptions of the plant growth parameters and environmental measurement protocols.

The camera image exposure was controlled by locking manual settings, as described below. Image file format, lens distortion, and color correction were tested. The Holland Scientific RapidScan CS-45 product (Holland Scientific, Lincoln, NE, USA) was used to acquire RED, Red-Edge (RE), and NIR spectral components at 670, 730, and 780 nm wave lengths (10 nm wide), respectively. The active reflectance values were collected concatenate with images to provide a camera-independent measurement of the plants’ optical signature. The collection of three spectral bands enabled the calculation of both Normalized Difference Vegetation Index (NDVI) and Normalized Difference Red-Edge (NDRE). While these indices are related, NDVI is more commonly used to assess canopy cover. However, NDVI can saturate at higher leaf area index values, potentially limiting its effectiveness in dense vegetation. NDRE is considered more sensitive to plant nitrogen and chlorophyll status, because it incorporates information from the spectral inflection point along the RE region [106,107,108].

Spreadsheet data were managed using MS Excel Version 2401 (Microsoft Corporation, Redmond, WA, USA), and statistical analysis was conducted in JMP 15.2.0 (SAS Institute, Cary, NC, USA) software, with a permutation ANOVA technique [109] implemented in the R programming language (R Core Team 2021) [110] as aovperm from the permuco 1.1.3 package [111].

**Figure 1:**
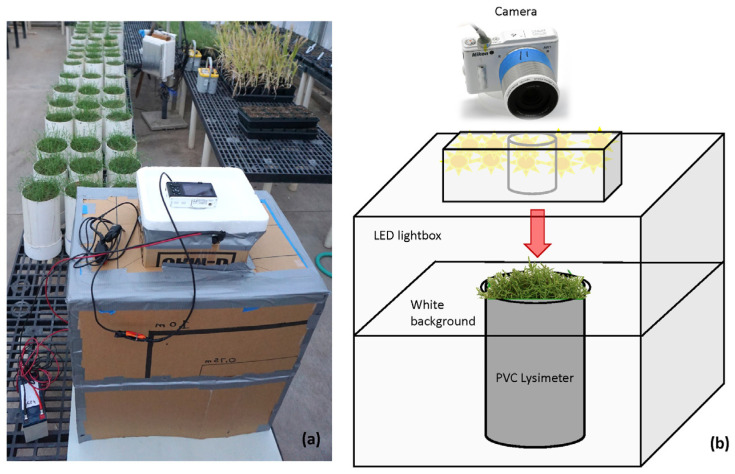
The lightbox is shown in greenhouse #1 (panel (**a**), left side) with the camera installed on top. The remote trigger with switch and the 12-volt power supply with 7.5 Ah SLA battery and wires are visible on the left and bottom left side. The lightbox diagram (panel (**b**), right side) illustrates the placement of LED lights and demonstrates how a lysimeter would be inserted into the box and photographed against the white background.

The custom lightbox design (Figure 1) was based on a modification of the foundational 2001 approach of Richardson and Karcher [92,93], and the 2016 design of Zhang et al. [94] that incorporates LED lights, each of which employs a portable photographic studio for digital image-based phenotyping of turfgrass. The rudimentary prototype lightbox used in this paper measured 57 (L) × 40 (W) × 74 (H) cm. It was constructed from a 0.5 cm thick repurposed cardboard, assembled with tape, and equipped with an internal horizontal image backdrop that fit flush around the 16 cm diameter lysimeter top (182.4 cm^2^). The camera was positioned with a lens protruding into a 24 (L) × 24 (W) × 14 (H) cm upper box containing LEDs. The camera lens was 27 cm distant from the lysimeter and pointed downward from the top of the box through an 11 (L) × 20 (W) × 6 (H) cm polystyrene camera cradle with a 7 cm hole that was 6 cm deep to fit the form of the lens. White printer poster paper was used as a generic, hue-neutral light reflector to cover the inside surfaces of the box. The box was wide enough so that the camera view of the lysimeter top and background would not extend to its sides; therefore, only the lysimeter top and white backdrop were in camera view. For each image sample, the box and one lysimeter were placed on a 60 (L) × 55.5 (W) cm light-colored paper poster board, which was positioned on the greenhouse bench to provide a consistent material foundation.

Photographic illumination was provided by an inexpensive generic 12-volt light emitting diode (LED) strip (Desvorry branded) consisting of 300 individual 6000 K white color lights. The light strip was affixed to the top of the box inside the perimeter around and just above the camera lens. Many LED lights were used for illumination to support light diffusion and reduce shadows. Light emission was measured by a pyranometer positioned at the height of the lysimeter top inside the lightbox. The low-cost light source proved inadequate for sustained use. It decreased in intensity from 16 to 8 watts m^2^ over 360 s, as described by Equation (1),

(1) $$Radiation=0.00005\times {Seconds}^{2}-0.0309\times Seconds+16.494$$

(coefficient of determination, R^2^ = 0.95) with a steady state of 8 watts m^2^ after 360 s (Standard Deviation, SD = 0.537). Changes in lighting during LED activation were likely due to temperature effects as the LEDs warmed rapidly. Therefore, the LEDs were only activated immediately before each lysimeter image sample and then turned off to mitigate fluctuations in brightness. The approach reduced measured illumination variance, resulting in an average of 16 watts m^2^ (SD = 0.482) across twelve test runs. Each test run consisted of 7 to 15 s of illumination with 60 s of de-energized time in between. This scenario was representative of the typical timing for lysimeter data collection of just over one minute per lysimeter.

The camera was set to “manual” mode for both acquisition and exposure, no exposure bias, no flash, and “pattern” focus metering. “Standard color” was selected with no “Active D-lighting”. The camera exposure was set to 160 ISO with an aperture of F-Stop 3.5 and a shutter speed of 1/320 s. An 11 mm focal length (equivalent to 29 for a 35 mm crop factor) was used, and the adjustable zoom lens was taped in position. A custom white balance was sampled and recorded on the camera by using the white poster paper placed inside the box and illuminated by the LEDs. The camera viewfinder displayed a circular graphic that was about the same size as the lysimeter top when visualized on the screen. This feature was utilized to verify straightness of camera angle and support geometric consistency between images. The camera display helped verify each time an image was taken and ensured that no settings were accidentally changed between image collections. A custom remote trigger was used to manually activate automatic focus and acquire images. High-resolution 4608 × 3072-pixel JPEG and 4548 × 3012-pixel Nikon NEF (RAW) image file formats were obtained (approximately 5.9 and 12.3 MB in size, respectively). This equated to a measurement resolution close to 11 pixels per mm. Three 24-bit sRGB standard color space image replicates and one RapidScan composite reflectance measurement were taken consecutively for each lysimeter sample. Average values obtained from the imagery were used for comparison with the individual VQ and NDVI lysimeter point measurements. Lysimeters were manually imaged in the morning, approximately every other week for 8 weeks. Chemical ice packs wrapped in microfiber cloth were employed to cool the camera between pictures and avoid overheating. Care was taken to position grass leaves above the imaging backdrop and in view of the camera and any loose leaves were removed from the imaging backdrop when changing lysimeters. Image processing was conducted on a Dell Latitude 5420 laptop with an i7-1185G7 1.8 GHz CPU and 16 GB of RAM (Dell Technologies Inc., Round Rock, TX, USA) running Microsoft Windows 11 Pro 22H2 64-bit OS (Microsoft Corporation, Redmond, WA, USA). This computer represents a typical consumer-grade product, and with the Python 3.12 software (Python Software Foundation, Beaverton, OR, USA) [112] that is commonly used in academic settings. Jupyter Lab and Notebook application tools (Project Jupyter, New York, NY, USA) were run using an Anaconda 2.5.0 installation (Anaconda, Inc., Austin, TX, USA) and included the Pandas, Numpy, Scipy, OpenCV, PIL, Scikit-Image, Matplotlib, and Seaborn libraries. When RAW imagery was used in the Python process, files were initially converted to TIFF (Tagged Image File Format) without compression using Irfan Skiljan’s IrFanView 4.59 (IrFanView, Lower Austria, Austria), which resulted in a file size of 40.1 MB. All imagery was converted to an 8-bit memory depth when processing in Python to reduce computational overhead. An 8-bit image can still display up to 16.7 million colors using 0–255 value tuples. It took approximately 60 s to process a single image file on the small laptop computer, regardless of the file type.

Color correction was used to ensure color accuracy [113] and was performed before application of lens distortion correction using the PerfectColor 1.0 program from Evan Lund of Evens Oddities (https://www.evenlund.com/, accessed on 19 October 2023). This program was freely available and allowed the batch correction of images using the 2014 standard X-Rite Color Checker Classic (24) Legacy panel. It used lookup tables (LUT) generated from a camera profile reference image of the color checker panel that was captured inside the lightbox. The PerfectColor 1.0 software is an easy-to-implement correction option. Color checker reference values are provided by Babel Color (https://babelcolor.com/, accessed on 6 September 2023), and RawTherapee 5.9 was used to sample and compare color values using its Lockable Color Picker tool. Image color values were also measured using ImageJ 1.54c for reference and comparison [114]. ImageJ is an open-source Java program credited to Wayne Rasband and contributors in association with the National Institutes of Health, USA. The software offers powerful image processing and analysis functions.

Lens distortion correction was performed after color correction using Python Discorpy 1.6.0 and following the tutorial at https://discorpy.readthedocs.io/en/latest/usage/demo_01.html, accessed on 13 April 2024, which can correct radial and perspective distortion by utilizing a grid pattern reference image. Graph paper was photographed inside the lightbox to provide the input pattern. The software calculated polynomial correction coefficients specific to the camera setup. Correction results showed the linear grid uniformity of graph paper control images upon manual review. Corrections were batch applied to the experiment sample images. Separately, lens vignette correction was performed in RawTherapee 5.9 using the settings of 48 strength and 45 radius for JPEG images, and 45 strength and 45 radius for TIFF images.

**Figure 2:**
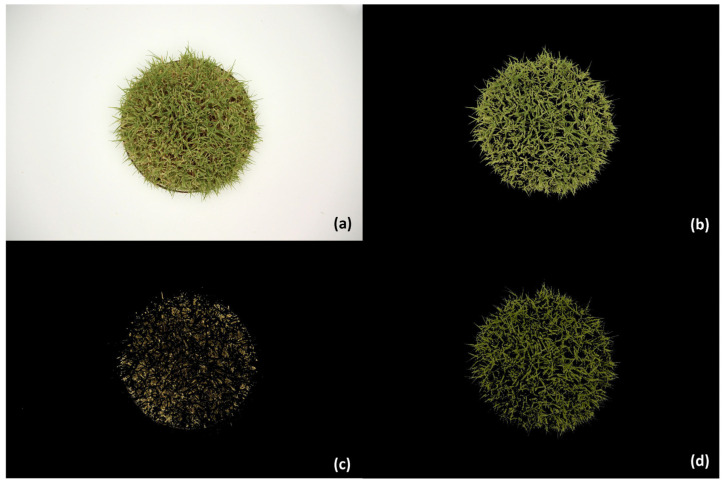
An example lysimeter uncorrected image and three masked views. Experiment treatment 30% water and 5.0 cm mow height is shown in an image taken on 10/26/2023 (Week 2) with associated 0.61 NDVI and 7.0 VQ (panel (**a**), upper left), 97.8% of the lysimeter area covered in live green material (%C) segment (panel (**b**), upper right), resulting in 0.280 DGCI, 0.400 HSVi, and 7.010 COMB2 calculation values, with 31.1% yellow (%Y) plant cover (panel (**c**), lower left), and 59.0% green (%G) cover fractions (panel (**d**), lower right).

Color classification areas were determined based on a pixel value-based binary mask used to segment regions of interest in the original image (Figure 2). The selected classification areas were analyzed as fractions of the total image area. They represented a chosen range for the total living plant material cover and the percentage of yellow senescent or chlorotic plant material, and a generic green color plant material range. Details are provided below and in Table 1. The bespoke living plant material cover segment was used to calculate discrete image color qualities for that fraction. Individual color scalar components were generated across several color spaces. Many calculations were made to evaluate vegetation indices from existing literature, and custom relationships were also formulated based on pervious linear correlation in separate imagery with NDVI and green cover area. Due to the large number of different calculated terms evaluated (280), only the most relevant metrics that best correlated with VQ and NDVI were selected to report in this paper. Although NDVI was collected at the same time as image metrics, VQ was not. There was an average difference of 2.2 days (SD = 0.92) between the assessment of VQ and the collection of image metrics.

The RGB color space is common in imagery [115], where the R term indicates the intensity of red colors, the G term indicates the intensity of green colors, and the B term indicates the intensity of blue colors. The three terms added together indicate a brightness value. The Hue–Saturation–Value (HSV) color space is derived from RGB values. It separates the color components into a single angle of Hue (H) ranging from 1 to 360 (or another value range of different graduation). Saturation (S) indicates the linear lightness of the Hue color, and Value (V) indicates the linear brightness. Saturation and Value are typically measured on a scale of 0–100 (0–1, or 1–255 for 8-bit imagery). A Value of 0 represents black, while a Saturation of 0 indicates gray without any color. Percent living plant cover classification segmentation (%C) was derived using the HSV color space. Where Hue (on a scale of 0–179) ranged from ≥27 to ≤90, Saturation (on a scale of 0–255) ranged from ≥60 to ≤255, and all brightness Values were included (on a scale of 0–255). The generic green fraction (%G) included Hue ≥ 30 and ≤60. Green fractional cover was previously reported by Casadesus to support wheat research [116]. Yellow area classification (%Y) involved Hue ≥ 16 to ≤26. Both %G and %Y used the same Saturation and brightness Value designations as %C (Table 1).

The Python-based image analysis used in this paper was modeled on the approach exemplified in the TurfAnalyzer 1.0.4 (TA) software. TA is a freely available software tool developed by Douglas Karcher in collaboration with Pennington Seed, Inc. (Madison, GA, USA), and Nexgen Turf Research LLC. (Albany, OR, USA) [117], based on the progressions of Karcher and Richardson [35,93] who originally used SigmaScan macros to measure turfgrass. It enables the analysis of JPEG images for percent green cover and dark green color index (DGCI) [118] and has been used in many experiments [119,120,121,122,123,124,125]. The default color values for TA were included in the Python analysis. These values were determined by converting the full Hue range of 1–360 and the Saturation and brightness Value ranges of 0–100 in TA, to a Hue range of 0–179 and Saturation and brightness Value ranges of 0–255 for the OpenCV commands, respectively. To better understand the computation process and verify data quality, calculated image values such as mean cover area classifications and DGCI were compared between the two software outputs and found to be equal. However, the more intricate Python approach was selected in this research due to a desire for enhanced process control, the capability to conduct full-resolution computations on TIFF format files, the option to export individual pixel values if wanted, the ability to export additional statistical values for each image, the need for additional calculations involving customized relationships between metrics, and the option to visualize and export individual values, histograms, or index calculations as separate charts.

A comprehensive range of Hue angles was selected for the fraction of living cover area (%C) classification segment, to include lower quality yellow-green living plant material through the range of green color to higher-quality green living plant material and into any blue-green material (although no blue-green plant material was present). This broad range was chosen to be inclusive of all the living plant material. Selection of the generic green color area (%G) centered around hue angle 45 and was intended to select only healthy vegetation with high chlorophyll and nitrogen contents. The fraction of senesced yellow plant material (%Y) selection included color values below those of the %C, but also excluded the shades of red and brown colors which indicated soil (the threshold was positioned between the color overlap of brown leaves and brown soil). The three classified color fractions included all brightness Values but used a Saturation threshold of 24% to ensure that selected pixels contained a color intensity which excluded the white background and bright reflection points. These classification parameters were derived from an iterative process of manual selection and evaluation detailed in the Supplemental text.

Figure 2a displays the original 2D view image sample for a 5 cm mow height and low water treatment. The hue-neutral white background is adjacent to the lysimeter perimeter, with grass leaves extending outward. Figure 2b depicts the HSV-based primary color segmentation of all living plant cover area. This comprehensive range of yellow-green to blue-green %C classification accurately includes all illuminated living plant material and was used as the basis for determining color qualities in subsequent calculations. Figure 2c shows the %Y fractional area of senescence with plant relevant Hue angles that are below the %C parameter but above the soil. Figure 2d shows the %G cover segmentation of healthy plant material.

This paper evaluated Guijarro’s RGB-based COMB2 calculation from [126], where three vegetation indices were combined for a model intended to be more sensitive to vegetation changes and less affected by soil background. It uses the Excess Green Index, which is designed to enhance detection of green vegetation, Equation (2):

(2) ExG=2×g–r−b

from [127], where r, g, and b values are normalized red, green, and blue; the Color Index of Vegetation Extraction index is designed to enhance green color for vegetation segmentation, Equation (3):

(3) CIVE=0.441×Red−0.911×Green+0.385×Blue+18.78745

from [128]; and the Vegetation Extraction Index is designed to emphasize green reflectance, Equation (4):

(4) $$VEG=\frac{g}{(r^{0.667})\times \left(b^{\frac{1}{0.667}}\right)}$$

from [129], where r, g, and b values are normalized red, green, and blue, and the alpha value of 0.667 was taken from [130] as a generic camera factor. These indices are combined to create Equation (5):

(5) COMB2=0.36×ExG+0.47×CIVE+0.17×VEG

Color spaces beyond the standard RGB and HSV were used. CIE 1976 Lab* (CIELAB) values were calculated with OpenCV, and individual color and brightness scalars were evaluated separately, as well as in relation to each other using various equations. The CIELAB coordinate system was adopted by the International Commission on Illumination (CIE) in 1976 [131] as a color space with increased uniformity relative to human visual perception [132,133]. The CIELAB color space is useful in many applications [134,135,136,137,138] with colors that use the standard observer model for device independency. The CIELAB color space achieves increased human perceptual uniformity because equal Euclidean distances in the color space correspond to equally perceived color differences. The CIELAB L* term represents lightness, but essentially, the a* term represents a green-to-red color dimension axis, and the b* term represents a blue-to-yellow color dimension axis. This paper uses the color opponent axis ratio of b* to a* (BA), intended to emphasize plant health indications of green and yellow pigment status, and the authors are not aware of other phenotyping papers using this ratio, although Wanono [139] investigated the ratio of a* to b* to detect leaf nitrogen. A higher BA ratio could signify chlorosis, senescence, or water stress, while a lower value could indicate presence of chlorophyll, nitrogen or increased water use efficiency.

An additional image-based equation was created to compare with standard DGCI. The unconventional Hue difference illumination ratio, or HSVi, is shown in Equation (6):

(6) $$HSVi=H-\left(\frac{S}{V}\right)\times 40^{-1}-0.3$$

This equation utilizes the HSV color space, where the simple ratio of Saturation and Value was subtracted from Hue, and the result was shifted and offset to scale roughly into a range of 0 to 1. By considering the Saturation-to-Value ratio, we capture a relative color intensity, and so as brightness increases, the Saturation impact reduces. A higher ratio indicates more vivid color relative to brightness. This nuanced HSVi approach modulates Hue angle as a function of color purity and attenuates the positive effect of Hue using lighting to discriminate minor plant features. Therefore, context is important for interpretation, because a larger HSVi result may indicate healthy but less dense vegetation with higher reflectance, while lower values may indicate stress or the presence of very dark green vegetation. The HSVi approach offers a color evaluation related to DGCI, but by subtracting the color purity to brightness ratio it could also help resolve more subtle or mixed canopy features.

Calculated metrics were summarized in tables and related to the experimental class structure. To test the effects of experimental treatments, response variables were assessed using a robust permutational multivariate analysis of variance (permANOVA). Non-parametric permutation ANOVA models are useful in environmental analysis to better handle complex data [140,141,142]. Neither the VQ nor the NDVI data in this paper use camera technology. Therefore, VQ data reported previously and NDVI measurements were chosen as independent references used to compare with the image-based data [36,143,144]. An NDVI time series overview for the original experiment is presented for the first time in this paper, and time series data for two image-based color metrics are offered to show comparative differences in behavior over the course of the experiment.

Image-based metrics were computed and compared with reference measurements to test the effects of file format with standard JPEG compression (JPG), NEF (RAW) to lossless TIFF conversion (TIF), and image adjustments for geometric lens distortion correction (LC) and color correction (CC). Pearson’s correlation coefficient (r) and coefficient of determination (R^2^) values were calculated, and the image metrics that best corresponded to each reference measurement were selected for reporting. A sensitivity analysis of calculated metrics was conducted to assess the impact of file format and image corrections. Significant differences were tested and a comparison of treatment effects on image metrics, VQ, and NDVI was performed using non-parametric Kruskal–Wallis median test and Dunn’s ranked test with JPEG as control. In a similar approach, the coefficient of variation was evaluated to investigate the effects of file format and image correction on replicate images. The authors are unaware of other published works detailing the sensitivity of file format and image corrections (JPEG, TIFF, LC, and CC) on phenotypic image calculations for turfgrass phenotyping.

## 3. Results

The %Y quantifies a pixel-based segment of percent cover across the lysimeter top area for yellow-colored senescent plant material. Lower values of yellow can indicate healthy and higher quality turfgrass. The %G area pixel-based fraction indicates presence of healthy plant biomass. DGCI describes the color of turfgrass, where higher values indicate a darker green turfgrass with increased chlorophyll pigments [145]. The HSVi calculation is akin to DGCI as it also describes plant color. HSVi is nuanced however, as higher values can indicate a higher quality of green for turfgrass, but context is important for interpretation since it is intended to capture subtle variations in vegetation density, stress levels, and chlorophyll content. The BA ratio of the b* and a* color scalars also represents a nuanced relationship where higher values indicate more yellowness relative to redness and lower values indicate more blueness relative to greenness. Therefore, pigmentation composition or environmental response may be detected with BA. These image metrics were selected because they showed linear associations with reference measurements and are either traditional plant health indicators or are new calculations which may offer additional insight into plant health.

### 3.1. Linear Correlation between Variables

Linear correlation and percent variance explanation were analyzed for the image calculations that related strongest to the reference measurements. This involved the continuous variables visual quality (VQ), grass clipping production (mg d^−1^), water use (mm d^−1^), NDVI, NDRE, NIR, RED, and RE (Table 2).

Results show that %Y cover area correlated most strongly with VQ, the calculation of DGCI correlated strongest with clipping production, COMB2 correlated most strongly with water use, %G correlated strongest with NDVI as well as RED, BA correlated most strongly with NDRE, and RE, and HSVi correlated strongest with NIR. The presence of correlation between image calculations and measurements with correlations coefficient absolute values greater than 0.8, 0.6, and 0.3 would indicate strong, moderate, and weak linear relationships, respectively, while the respective coefficient of determination values above 0.8, 0.6, and 0.3 would indicate a strong, moderate, and weak explanation of variance. The correlations presented illustrate how imagery data can be useful to resolve phenotypic responses [105]. However, there was no clear trend in the correlations regarding effects of the image corrections or file format.

Data suggest that selected image-based calculations correlate with VQ, clipping production (mg d^−1^), water use (mm d^−1^), and spectral reflectance values. However, the image format and correction process only sometimes improved resultant correlations. The uncorrected TIF and the JPG LC for %G classification area exhibited the strongest correlation with NDVI and RED, respectively, but there was a nominal 0.000 and 0.001 respective difference in R^2^ between the uncorrected TIF and the JPG LC with JPG for each. The relationship between BA with NDRE, and RE showed the strongest correlation, but R^2^ only improved by 0.004 for NDRE and 0.003 for RE over the uncorrected JPG. %Y and VQ were similar, where the JPG LC CC showed the strongest correlation, but the R^2^ was only 0.005 higher than the uncorrected JPG. DGCI TIF CC correlated best with productivity, but the correction showed only minor differences in R^2^ with a 0.098 increase. Likewise, the HSVi TIF CC correlated the strongest with the NIR but the R^2^ only increased 0.014. One metric, COMB2, exampled a big improvement with TIF LC CC. The COMB2 had the strongest correlation with water use where it exhibited an R^2^ value 0.310 higher than its uncorrected JPG counterpart. Therefore, it would be improbable to apply none, all, or any individual correction procedure and consistently achieve the highest correlation results across this variety of specific image-based calculations. When correction was optimized, the %Y showed a 0.17 higher R^2^ value than DGCI for VQ, %G showed a 0.07 higher R^2^ value than DGCI for NDVI, HSVi showed a 0.09 higher R^2^ than DGCI for NIR, and BA showed a 0.06 higher R^2^ than DGCI for NDRE, suggesting that the DGCI calculation did not always explain the most variance in the reference measurements regardless of correction.

### 3.2. Effects of Image Corrections on Calculated Metrics

The TIFF file format and three correction types were compared against the uncorrected JPEG format to assess their impact on median values for six image-based metrics as shown in Table 3. Results are mixed regarding the significant effects and the magnitude of the correlations with reference measurements. There was a significant difference among all the median values of calculated metrics, except for %Y, even though the JPG LC CC shifted the median value of %Y by 1.6%. TIF format without correction shifted the %G median by 3.7%, but it was not significant. Similarly, JPG LC shifted the BA median by 0.9%, but it was not significant. However, TIF LC did significantly shift medians (24%) for DGCI and HSVi, by 0.075 and 0.102, respectively. Most striking was the effect of corrections on COMB2, where TIF LC CC significantly shifted the median by −13.97 (or 133.7%). Corrections for four of the six metrics that resulted in the highest or lowest median values did not correlate the strongest with their associated reference measurements. However, DGCI TIF CC resulted in the highest median value, and it correlated most with productivity (Table 2), while COMB2 TIF LC CC resulted in the lowest median value, and it correlated the strongest with water use (Table 2). Median values did tend to improve along their relative scales with CC. However, there was no clear trend across all the metrics. Therefore, these data support the idea that neither the file format nor any specific image correction consistently improved model power for these image-based calculated metrics.

### 3.3. Detection of Experiment Treatment Effects

A robust non-parametric permutation-based ANOVA was chosen to handle the skewed and complex environmental variables. Results indicate the effect sizes and likelihood of significant treatment effects on image metrics and reference measurements. Mowing height had a significant impact on all variables and was primarily explained by VQ. The irrigation effect was significant for all variables and was also primarily explained by VQ. All variables were significantly affected by Date, where the HSVi and DGCI prominently explained the Date effect, followed by BA. A substantial portion of the overall effect size for NDVI, %G, and %Y was also attributed to the Date, indicating the detection of the autumn seasonal trend towards biological dormancy. The interaction between mowing height and water was significant for all variables except DGCI and was best explained by COMB2, although this interaction category had the most unexplained variance overall. The interaction effect of mowing height by date was significant for all variables except VQ and DGCI and was best explained by COMB2 with a reduced but still large effect size. The irrigation-by-date interaction was significant for all variables except DGCI and HSVi, where VQ provided the best explanation followed by %Y. The three-way interaction effect was significant for all variables except VQ and %G, but was explained most by COMB2, indicating the most sensitivity to experimental treatment interactions with this combination index variable.

These results suggest that the different metrics play distinct roles in resolving various aspects of the biological information signal. The mean values were significantly different for all treatment effects, suggesting potential utility for detecting variations in irrigation quantity, mowing height, changes over time, and their interactions. The COMB2 variable may have outperformed NDVI with higher categorical effects significance, but the ηp2 (and F-values) were highest for %Y. The reduced effect sizes for the interaction of mowing height and irrigation suggest that the dynamics of this treatment merit further resolution. Additional tables of the *p*-values and F-values used to create Table 4 are provided (in the Supplementary Materials) to offer more precise probability values of significant differences and additional model fit measures.

### 3.4. Image Corrections Effect on Replicates

Three replicate images were initially taken in rapid succession for each of the 316 lysimeter samples. A coefficient of variation (CV) was calculated for each image metric and with each image correction, and the CV values were averaged across all collections for each file type and correction. The average values of CV were compared to the correlations (Table 2) and ANOVA (Table 3) results. The CV values are presented in Table 5.

Significant differences in image correction effects were observed in the average CV values between replicated images for all metrics except %Y and BA, using the Kruskal–Wallis test of medians and Dunn’s test with JPG as control. The JPG LC CC for the %Y correlated strongest with VQ (Table 2), even though TIF LC CC had the lowest CV. The %G TIF data correlated best with NDVI and RED reflectance (Table 2), even though TIF LC CC had the lowest CV and a noticeable difference for CC with reduced CV was evident. The DGCI TIF CC most strongly correlated with productivity (Table 2), even though the uncorrected JPG and TIF formats showed lower CV values and there was an increased CV with LC visible. HSVi calculation in TIF CC imagery correlated the strongest with NIR reflectance (Table 2), even though the TIF and JPG had lower CV values and a general increase in CV with CC was visible. The JPG LC BA calculation most strongly correlated with NDRE and RE reflectance (Table 2), even though the uncorrected JPG had the lowest CV. The COMB2 calculation of JPG LC imagery correlated the strongest with water use, even though the uncorrected TIF had the lowest CV and there was generally a higher CV with corrections.

Image corrections significantly affected the median CV of image replicates in four out of the six metrics. However, the median CV of the image replicates for each calculated metric did not explain the subsequent model fit. Higher values of CV did not result in reduced explanatory capacity. Yet the uncorrected JPEG file format showed a CV lower than the overall average for each metric. These results demonstrate a weak relationship between the dispersion in replicates and subsequent correlation with reference measurements. Therefore, the median dispersion of image values from replicates did not indicate a better model fit or better explain the reference measurements for these six calculated metrics. The analysis of CV also did not consistently identify a specific image format or correction method that led to higher or lower data dispersion. This result supports the notion that the effects of the image corrections applied are present, but they are difficult to parameterize and explain across multiple and varied image-based calculations.

### 3.5. Time Series Charts for Three Metrics, NDVI, %Y, and COMB2

**Figure 3:**
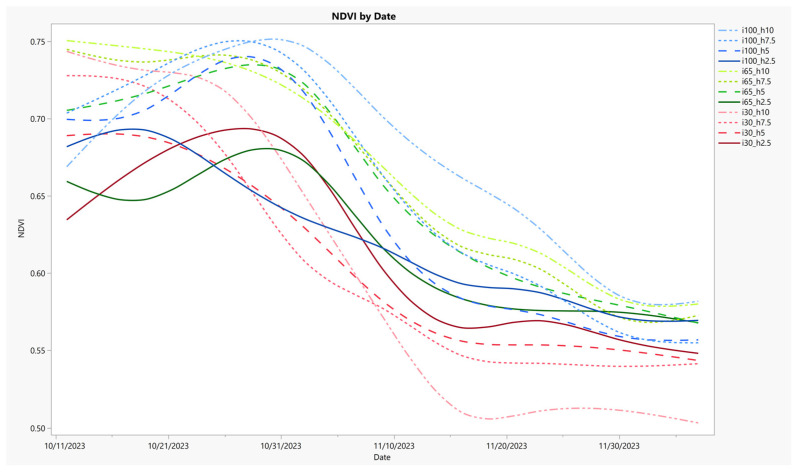
NDVI time series chart with NDVI plotted on the Y-axis and date on the X-axis. The experimental treatments are labeled by their percentage of consumptive demand-based irrigation supplied (i = 100, 65, and 30) and their mowing heights (h = 10, 7.5, 5.0, and 2.5 cm). Each treatment is grouped by irrigation level and is uniquely colored, and the line pattern is based on mowing height. NDVI shows changes in time and differences with experimental treatment.

NDVI was not previously reported by Hejl [105] and is shown here (Figure 3) to illustrate the utility of this common measurement, which was used as a reference for evaluating the imagery data in this paper. Treatments are displayed in a time series throughout the autumn season experiment. A general downward trend in NDVI was evident, but several treatments exhibited increased NDVI over the first third of the experiment before decreasing thereafter. At the beginning of the experiment, the shorter mowing heights automatically had decreased presence of green biomass, resulting in lower NDVI values compared to taller heights. As the experiment progressed and the irrigation deficit treatment intensified, shorter mowing heights were able to sustain their green biomass presence longer. In the lowest irrigation treatment, the relative NDVI values ranged from largest to smallest, corresponding to the heights from shortest to tallest. The middle irrigation treatment also exhibited this effect, but with less pronouncement, where NDVI values became comparable by the end of the experiment. Full irrigation treatment exhibited the highest NDVI values overall, and taller mowing heights were more effective in preserving green biomass when given adequate water. The NDVI values for the fully irrigated plants were generally arranged from taller to shorter heights, except for the shortest height, which had the second highest NDVI values and was comparable to the tallest full irrigation treatment. Results suggest that green biomass presence may be prolonged under reduced water availability with shorter mowing heights. Additionally, a moderate reduction in water availability may have a limited effect on NDVI regardless of mowing height. However, the largest green biomass as measured by NDVI can be expected with higher mowing heights and full demand replacement water applications.

**Figure 4:**
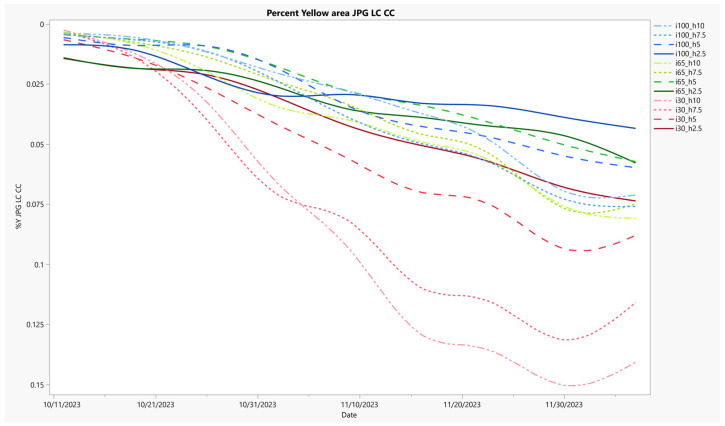
A %Y time series chart is presented, where the image-based yellow color classification segment is plotted on the inverted Y-axis and the date is on the X-axis. The experimental treatments are labeled by their percentage of consumptive demand-based irrigation supplied (i = 100, 65, and 30) and their mowing heights (h = 10, 7.5, 5.0, and 2.5 cm). Each treatment is grouped by irrigation level and uniquely colored, the line pattern is based on mowing height. Results show change over time and increased treatment separation with the greatly reduced water treatment.

VQ was previously reported by Hejl in 2024 [105]. Therefore, a time series of the senesced plant indication %Y area segment is shown as a proxy relating to the experimental treatments, as calculated from the JPG LC CC image set (Figure 4). The %Y had the highest correlation with VQ, an R^2^ of 0.79 (Table 2) and demonstrated the highest effects size overall (Table 4). The %Y was scaled as a percentage of the top inside area of the lysimeter. Values could exceed 100% if yellow-colored leaves covered the lysimeter area and extended beyond the perimeter of the lysimeter top. The classification of the %Y showed a more consistent trend than the NDVI, basically increasing for all treatments across the experimental period. However, shorter mow heights generally resulted in less yellow presence. Unexpectedly, the 7.5 cm mow height showed more %Y than the 10 cm height for both the highest and middle irrigation levels indicating a possible microclimate, root system, or nutrient allocation impact. Results suggest that the image-based classification of the fractional yellow area can be an effective metric for assessing visual quality in turfgrass. It can also differentiate the mow height effect when water is significantly reduced.

**Figure 5:**
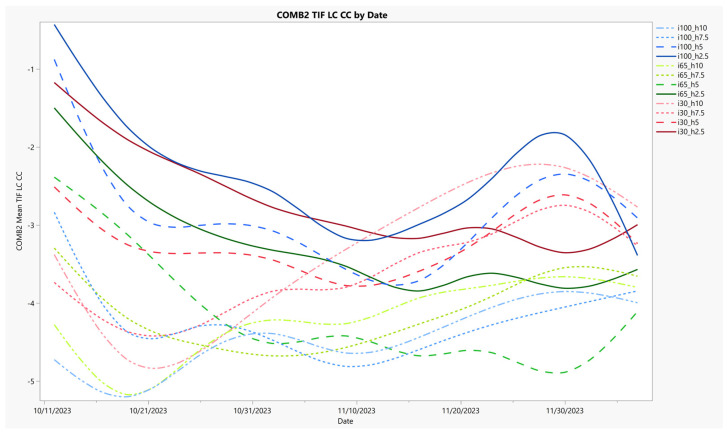
A COMB2 time series chart is presented where the combination term is plotted on the Y-axis and date is on the X-axis. The experimental treatments are labeled by their percentage of consumptive demand-based irrigation supplied (i = 100, 65, and 30 actual evapotranspiration replacement) and their mowing heights (h = 10, 7.5, 5.0, and 2.5 cm). Each treatment is grouped by irrigation level and uniquely colored, the line pattern is based on mowing height. Results show reduced change over time, but increased treatment separation when compared to NDVI and %Y (Figure 3 and Figure 4).

COMB2 data are presented in time series (Figure 5) to show the different behavior of this color metric over time, as compared to NDVI and %Y, because it most correlated with water use (Table 2), and because it showed significant difference for all experimental effects (Table 4). A complex and nuanced response is evident using this combination of vegetation indices. Higher COMB2 values suggest healthier vegetation and more biomass. This can be seen in the first half of the experiment where values decline as plants tended to decrease in quality over the experiment. However, these results are difficult to interpret because unlike NDVI and %Y (Figure 3 and Figure 4), there are larger COMB2 initial treatment differences that converge as the experiment progressed. This may indicate the effect of season and the lowest irrigation, where the two taller mow heights increased in COMB2 value over the experiment even though their color quality decreased, perhaps as a response to change in canopy density. Likewise, the initial separation regarding mow height groupings that is evident across the irrigation levels may suggest distinct canopy structures. Another consideration is that the image correction effect was very large for this combined index metric (Table 3), where different corrections substantially changed the correlations with the different reference measurements (Table 2). Nevertheless, the statistical significance with treatment effects results suggests an information contribution from the COMB2 color term with TIF LC CC application, where the effect size for three out of the four treatment interaction terms was largest with COMB2.

Additional time series charts for the metrics of %C, %Green, DGCI, HSVi, NDRE, NIR, RED, and RE metrics are provided in the Supplementary Materials.

## 4. Discussion

To assist researchers, students, and managers, the authors propose a practical approach that includes accessible camera controls for collecting relevant image-based plant phenotyping data. Locked manual photographic exposure settings that balance a lower ISO, moderately higher F-Stop, and, lastly, a higher shutter speed should be selected based on lighting intensity of the target environment. It is important to measure and set a customized white balance for the specific spectral intensity of the ambient illumination rather than a pre-set color temperature. It may be acceptable to rely solely on modern camera technology with simple exposure controls and achieve sufficient image data quality to perform basic plant phenotyping for advancement of scientific knowledge. However, in some instances, such as with the calculation of COMB2 in this paper, image corrections induced significant effects strong enough to change result interpretations. To overcome possible gaps in knowledge, transparent standardized protocols and detailed metadata are needed that include cross or independent validations and robust statistical analysis utilizing sample replicates and controls.

Utilizing a simple camera-based plant phenotyping approach, the irrigation and mow height experiment measurements from [105] were used as references for image calculations. Correlations between image calculations and reference measurements verify that a consumer-grade camera with exposure control can resolve phenotypic responses for turfgrass in a greenhouse, and how new calculations with increased correlations over standard metrics such as DGCI are possible. The highest R^2^ values were observed in the relationship between VQ with %Y area classification, and NDVI with %G area classification (Table 2). Traditionally, the evaluation of turfgrass has focused only on green color areas. However, our findings reveal that the presence of yellow, which demonstrated congruence with human perception of quality and significant treatment effects, offers insight into assessing the relative presence of green biomass in turfgrass. Casadesus [146] introduced a “Green” and “Greener” segmentation in wheat, incorporating some yellow into the “Green” classification. By introducing a dedicated yellow cover class, such as the %Y metric employed in our study, the yellow stems and senesced plant material is captured and quantified more comprehensively. A more complete classification system that includes the readily observable visual cue of yellow may allow for greater precision in evaluating the composition and health of turfgrass which could improve phenotyping analyses.

Further research could clarify COMB2 value in relation to plant biological status because the detected treatment effects significance with image corrections (Table 4) suggests a utility of this color calculation to support understanding of plant phenotypic traits, but COMB2 behaved differently than traditional terms like NDVI (Figure 3). Although the COMB2 calculation may provide some phenotypic detection capability and correlated the strongest with water use, it was not powerful enough to predict water use. Similarly, although DGCI correlated strongest with clipping production (Table 2), the relationship was not robust enough to explain most of the variation in that reference measurement. The BA ratio correlated most with NDRE and RE reflectance, while the novel term HSVi correlated most with the NIR spectral reflectance component (Table 2). Therefore, the calculation of BA or HSVi may be useful to roughly estimate respective RE or NIR spectral reflectance when only color camera data are available. The BA ratio may serve to emphasize yellowness relative to redness and provide an indication of chlorophyll content or plant health, but authors did not find reference to this ratio in the plant phenotyping literature so additional research is warranted. HSVi showed stronger correlation with NDVI than DGCI (Table 2). Therefore, variations in this illumination modulated Hue angle determined by HSVi may help detect pigmentation status or tissue health across the biomass area or over time. However, because authors are not aware of other research using the HSVi image calculation for plant phenotyping, additional research is needed to determine biological significance.

This paper demonstrates how image acquisition using consumer cameras with simple controls enables not only traditional metrics like the green area or DGCI but also facilitates additional calculations which may be valuable in the description of plant phenotypes such as bespoke yellow area, HSVi, or BA. Although it could be possible to avoid image corrections and still achieve acceptable results when simple exposure controls are used, the application of appropriate image corrections before the calculation of image metrics will likely generate the most useful information. The optimal combination of file format and image correction improved the correlations with reference measurements (Table 2) and significant treatment effects sizes and detections (Table 4). However, the varied and mixed correction effects results in this paper suggest further research is necessary to precisely identify correction effects on individual image calculations relative to phenotypic expression and prescribe the best methods to maximize corrections utility.

The findings in this paper show that the image corrections applied resulted in inconsistent outcomes. While correlations with reference measurements generally improved with image corrections, improvements were often minor and sometimes negative. Only %Y showed no significant effect from the TIFF file format or the image corrections, while CC did cause large significant differences in COMB2, BA, DGCI, and HSVi (Table 3). The mean coefficient of variation for replicates was significantly affected by corrections for %G, DGCI, HSVi, and COMB2 (Table 5). The optimal combination of file format and image corrections improved R^2^ values for five out of the six image metrics but resulted in less than 2% improvement. However, COMB2 was improved by 31% (Table 2). This suggests that the application of all standard image corrections may not automatically improve plant phenotyping results, and yet a lack of best file format and correction implementation could lead to the oversight of crucial relationships. An improved statistical evaluation using a larger and more diverse set of images and reference measurements, including the observation of additional plants beyond TifTuf over a longer period is needed to gain a deeper understanding of the image adjustments effects and inform best practices. Furthermore, the inclusion of more robust phenotyping equipment to provide thermal, hyperspectral, florescence, and structural information would better contextualize the potential of the traditional camera.

## 5. Conclusions

A consumer-grade camera and custom-fit lightbox made from rudimentary materials were used to verify the cost-effective method for plant phenotyping. Image-based metrics were calculated using open-source software on a laptop computer and enabled the comparison of human-assessed VQ and active NDVI measurements with image data to determine phenotyping utility. Statistical analysis revealed the highest R^2^ values for VQ and NDVI with the image-based calculations of %Y and %G areas, respectively. A novel HSV color space calculation, termed HSVi demonstrated the highest R^2^ with NIR, while the new CIELAB b* to a* ratio correlated strongest with NDRE and RE reflectance. Even though DGCI exhibited the highest R^2^ value for biomass productivity, it did not explain most of the variation. The COMB2 calculation showed significant experimental treatment effects, superior to VQ and comparable to NDVI but was difficult to interpret. COMB2 correlated the strongest with water consumption but was not adequate to serve as a proxy. %Y displayed increased overall treatment explanatory power for the experimental effects. Guidance on photographic exposure control was provided to improve the quality of image-based phenotyping data, along with a discussion on software options, processes, and limitations in Supplemental text [147,148,149,150,151,152,153,154,155,156,157,158,159,160,161,162,163,164,165,166,167,168,169,170,171,172,173,174,175,176,177,178,179,180,181,182,183,184].

This study indicates that modern camera technology with simple controls is robust enough to empower plant phenotyping for research and commercial applications that involve cover and color quantification. However, the imagery did not fully describe water use or clipping productivity. Despite testing color and lens corrections and the use of uncompressed image format, no large and consistent improvement in the image-based calculations were observed. This suggests that lossless format and corrections may not always be necessary for standard plant phenotyping when modern cameras, sufficient illumination, and straightforward exposure controls are utilized. A method to quantify the effects of image corrections relative to plant phenotype is needed to determine appropriateness for resolving plant traits of interest with sufficient precision. Parameterizing the influence of corrections on image-based calculations for different phenotyping metrics would strengthen the connection between photographic digital information and plant biological processes, standardizing and enhancing phenotyping practices.

Mention of a trade names or commercial products in this publication is solely for the purpose of providing specific information and does not imply recommendation or endorsement by the U.S. Department of Agriculture or any part herein. USDA is an equal opportunity provider and employer.

## Figures and Tables

**Table 1:** Color classification segmentation pixel value ranges used in the Python process.

	Hue	Saturation	Value
%C	27–90	60–255	1–255
%Y	16–26	60–255	1–255
%G	30–60	60–255	1–255

Hue is scaled to 0–179, and Saturation and Value are scaled to 0–255 for fractional living plant cover, yellow, and green as %C, %Y and %G, respectively.

**Table 2:** Person’s correlation coefficient (r) and coefficient of determination (R^2^) values for image metrics and associated reference measurements.

		VQ (316)		mg (280)		mm (108)		NDVI (316)		NDRE (316)		NIR (316)		RED (316)		RE (316)	
		r	R^2^	r	R^2^	r	R^2^	r	R^2^	r	R^2^	r	R^2^	r	R^2^	r	R^2^
%Y	JPG	−0.886	0.785	−0.463	0.214	−0.153	0.023	−0.804	0.646	−0.829	0.688	−0.798	0.637	0.723	0.522	0.695	0.483
	JPG CC	−0.889	0.790	−0.476	0.227	−0.138	0.019	−0.808	0.652	−0.836	0.699	−0.806	0.650	0.725	0.525	0.699	0.489
	JPG LC	−0.887	0.787	−0.463	0.214	−0.156	0.024	−0.804	0.646	−0.829	0.688	−0.798	0.636	0.723	0.522	0.695	0.483
	**JPG LC CC**	**−0.889**	**0.790**	−0.477	0.227	−0.143	0.020	−0.810	0.656	−0.838	0.702	−0.808	0.652	0.727	0.528	0.701	0.491
	TIF	−0.887	0.786	−0.464	0.215	−0.153	0.023	−0.804	0.646	−0.830	0.688	−0.798	0.637	0.723	0.522	0.695	0.483
	TIF CC	−0.889	0.790	−0.476	0.227	−0.133	0.018	−0.805	0.648	−0.836	0.698	−0.805	0.649	0.722	0.521	0.698	0.488
	TIF LC	−0.887	0.787	−0.471	0.222	−0.155	0.024	−0.810	0.656	−0.831	0.691	−0.801	0.642	0.731	0.535	0.696	0.484
	TIF LC CC	−0.885	0.783	−0.493	0.243	−0.131	0.017	−0.812	0.659	−0.833	0.694	−0.807	0.651	0.734	0.539	0.694	0.482
DGCI	JPG	0.744	0.553	0.600	0.360	−0.109	0.012	0.765	0.586	0.840	0.706	0.841	0.707	−0.666	0.443	−0.673	0.453
	JPG CC	0.785	0.616	0.608	0.369	−0.068	0.005	0.804	0.646	0.850	0.723	0.849	0.721	−0.717	0.515	−0.686	0.471
	JPG LC	0.757	0.574	0.581	0.338	−0.114	0.013	0.768	0.590	0.865	0.748	0.861	0.741	−0.655	0.429	−0.699	0.488
	JPG LC CC	0.793	0.629	0.604	0.364	−0.069	0.005	0.813	0.661	0.873	0.761	0.870	0.756	−0.718	0.515	−0.706	0.498
	TIF	0.738	0.545	0.599	0.359	−0.108	0.012	0.765	0.585	0.839	0.704	0.840	0.706	−0.666	0.444	−0.671	0.451
	**TIF CC**	0.786	0.618	**0.608**	**0.370**	−0.070	0.005	0.802	0.644	0.849	0.721	0.848	0.719	−0.716	0.512	−0.686	0.470
	TIF LC	0.508	0.258	0.419	0.176	−0.203	0.041	0.455	0.207	0.521	0.271	0.529	0.280	−0.387	0.149	−0.414	0.171
	TIF LC CC	0.753	0.567	0.591	0.350	−0.116	0.013	0.743	0.552	0.795	0.633	0.797	0.634	−0.658	0.433	−0.642	0.412
COMB2	JPG	0.613	0.375	0.471	0.222	−0.214	0.046	0.510	0.261	0.513	0.264	0.515	0.265	−0.469	0.220	−0.433	0.187
	JPG CC	0.073	0.005	0.121	0.015	−0.479	0.229	−0.126	0.016	−0.044	0.002	−0.022	0.000	0.160	0.026	0.028	0.001
	JPG LC	0.646	0.417	0.480	0.230	−0.016	0.000	0.660	0.436	0.624	0.389	0.626	0.391	−0.630	0.397	−0.519	0.270
	JPG LC CC	0.012	0.000	0.044	0.002	−0.580	0.337	−0.245	0.060	−0.085	0.007	−0.070	0.005	0.317	0.100	0.047	0.002
	TIF	0.616	0.380	0.471	0.222	−0.212	0.045	0.513	0.263	0.515	0.265	0.515	0.266	−0.471	0.222	−0.434	0.188
	TIF CC	0.015	0.000	0.078	0.006	−0.491	0.241	−0.177	0.031	−0.096	0.009	−0.072	0.005	0.206	0.042	0.072	0.005
	TIF LC	0.647	0.419	0.426	0.181	−0.082	0.007	0.614	0.378	0.633	0.401	0.624	0.390	−0.553	0.306	−0.530	0.281
	**TIF LC CC**	−0.052	0.003	−0.012	0.000	**−0.597**	**0.356**	−0.252	0.064	−0.107	0.011	−0.085	0.007	0.313	0.098	0.079	0.006
%G	JPG	0.739	0.546	0.460	0.211	0.401	0.161	0.911	0.829	0.863	0.745	0.849	0.720	−0.868	0.753	−0.705	0.498
	JPG CC	0.731	0.534	0.433	0.187	0.439	0.193	0.897	0.804	0.844	0.713	0.826	0.683	−0.857	0.734	−0.694	0.481
	JPG LC	0.736	0.542	0.460	0.211	0.405	0.164	0.911	0.829	0.862	0.743	0.848	0.719	**−0.868**	**0.754**	−0.705	0.497
	JPG LC CC	0.727	0.529	0.430	0.185	0.447	0.200	0.896	0.802	0.841	0.708	0.823	0.677	−0.857	0.734	−0.692	0.479
	**TIF**	0.738	0.545	0.460	0.211	0.403	0.162	**0.911**	**0.830**	0.863	0.744	0.848	0.720	−0.868	0.753	−0.705	0.497
	TIF CC	0.725	0.525	0.426	0.182	0.455	0.207	0.892	0.796	0.837	0.700	0.818	0.670	−0.854	0.730	−0.689	0.474
	TIF LC	0.733	0.538	0.462	0.214	0.415	0.173	0.904	0.817	0.851	0.724	0.838	0.702	−0.865	0.748	−0.696	0.485
	TIF LC CC	0.717	0.515	0.429	0.184	0.469	0.220	0.888	0.788	0.827	0.683	0.809	0.655	−0.854	0.729	−0.680	0.462
BA	JPG	0.829	0.688	0.554	0.307	0.084	0.007	0.862	0.742	0.902	0.813	0.888	0.788	−0.772	0.595	−0.734	0.539
	JPG CC	0.836	0.698	0.553	0.305	0.095	0.009	0.865	0.748	0.902	0.814	0.886	0.786	−0.776	0.602	−0.736	0.542
	**JPG LC**	0.826	0.682	0.552	0.305	0.088	0.008	0.863	0.744	**0.904**	**0.817**	0.890	0.792	−0.772	0.596	**−0.736**	**0.542**
	JPG LC CC	0.834	0.696	0.555	0.308	0.096	0.009	0.865	0.749	0.901	0.812	0.886	0.786	−0.778	0.605	−0.735	0.541
	TIF	0.827	0.683	0.555	0.308	0.084	0.007	0.862	0.744	0.902	0.813	0.888	0.789	−0.773	0.597	−0.734	0.539
	TIF CC	0.836	0.698	0.552	0.305	0.097	0.009	0.866	0.749	0.902	0.813	0.887	0.786	−0.777	0.604	−0.736	0.542
	TIF LC	0.774	0.599	0.554	0.306	0.045	0.002	0.798	0.636	0.821	0.674	0.816	0.666	−0.724	0.525	−0.665	0.442
	TIF LC CC	0.814	0.663	0.567	0.321	0.068	0.005	0.830	0.689	0.854	0.729	0.844	0.712	−0.753	0.566	−0.695	0.483
HSVi	JPG	0.747	0.558	0.514	0.265	0.192	0.037	0.881	0.776	0.898	0.807	0.891	0.795	−0.805	0.648	−0.716	0.513
	JPG CC	0.779	0.607	0.565	0.320	0.085	0.007	0.876	0.767	0.902	0.813	0.899	0.808	−0.795	0.632	−0.722	0.521
	JPG LC	0.758	0.574	0.543	0.295	0.135	0.018	0.882	0.778	0.898	0.807	0.895	0.801	−0.807	0.652	−0.714	0.510
	JPG LC CC	0.773	0.597	0.571	0.327	0.064	0.004	0.869	0.755	0.893	0.798	0.892	0.796	−0.790	0.624	−0.714	0.509
	TIF	0.742	0.551	0.514	0.264	0.190	0.036	0.881	0.776	0.898	0.806	0.891	0.795	−0.805	0.648	−0.715	0.511
	**TIF CC**	0.780	0.608	0.564	0.318	0.094	0.009	0.878	0.770	0.902	0.814	**0.899**	**0.808**	−0.798	0.637	−0.723	0.523
	TIF LC	0.682	0.465	0.512	0.262	0.103	0.011	0.754	0.568	0.758	0.575	0.759	0.575	−0.697	0.485	−0.602	0.363
	TIF LC CC	0.755	0.570	0.574	0.330	0.041	0.002	0.819	0.671	0.839	0.704	0.840	0.705	−0.748	0.559	−0.671	0.451

Bold font identifies which file format and correction that had the highest correlation with the image calculation’s reference measurement. The number of observations is noted in parenthesis beside the reference measurement in the top row. The file format and corrections applied are listed in the left column for each image metric.

**Table 3:** Medians and significant difference for file format and correction effects.

	%Y	%G	DGCI	HSVi	BA	ALI	COMB2
JPG	0.2652	0.8965	0.2960	0.4331	−3.5899	0.5614	10.4521
JPG CC	0.2485 ^NS^	0.9616 ***	0.3642 ***	0.5293 ***	−2.7051 ***	0.5637 ***	−2.5148 ***
JPG LC	0.2653 ^NS^	0.9305 ***	0.2787 *	0.4393 ^NS^	**−3.6054** ^NS^	0.5656 ***	16.5374 ***
JPG LC CC	**0.2494** ^NS^	0.9909 *	0.3571 *	0.5359 ***	−2.6843 ***	0.5681 ***	−3.3534 ***
TIF	0.2722 ^NS^	**0.9335** ^NS^	0.2917 ^NS^	0.4329 ^NS^	−3.5832 ^NS^	0.5613 ^NS^	10.4991 ^NS^
TIF CC	0.2568 ^NS^	0.9958 **	**0.3667** ***	**0.5351** ***	−2.6672 ***	0.3641 ***	−2.9138 ***
TIF LC	0.2788 ^NS^	0.9586 ^NS^	0.2872 ^NS^	0.4475 *	−3.4666 ^NS^	0.5676 ***	12.7586 ^NS^
TIF LC CC	0.2638 ^NS^	1.0121 ***	0.3637 ***	0.5503 ***	−2.5862 ***	0.5676 ***	**−3.5182** ***
Kruskal-Wallis	NS	***	***	***	***	***	***

Bold font indicates the correction for each metric variable which correlated best with its associated reference measurement (Table 2). Analysis utilizes a one-way Chi-square approximation of the non-parametric Kruskal–Wallis test and Dunn’s joint ranks test with Bonferroni adjustment using the JPEG file format as control. Significant differences in median values are noted as not significant, *p* ≤ 0.05, *p* ≤ 0.01, and *p* ≤ 0.001 using the symbols NS, *, **, and ***, respectively.

**Table 4:** Partial eta-squared (ηp2) effect sizes and significance level of permutational multivariate ANOVA.

			JPG_LC_CC	TIF	TIF_CC	TIF_CC	JPG_LC	TIF LC CC
	VQ	NDVI	%Y	%G	DGCI	HSVi	BA	COMB2
Mowing (M)	0.09 ***	0.02 ***	0.05 ***	0.04 ***	0.05 ***	0.00 *	0.02 ***	0.20 ***
Irrigation (I)	0.21 ***	0.06 ***	0.15 ***	0.11 ***	0.05 ***	0.06 ***	0.07 ***	0.05 ***
Date (D)	0.41 ***	0.66 ***	0.52 ***	0.57 ***	0.71 ***	0.72 ***	0.69 ***	0.15 ***
M × I	0.02 ***	0.02 ***	0.04 ***	0.02 ***	0.01 ^NS^	0.01 *	0.01 **	0.06 ***
M × D	0.02 ^NS^	0.03 **	0.06 ***	0.07 ***	0.02 ^NS^	0.03 **	0.03 **	0.15 ***
I × D	0.08 ***	0.03 ***	0.07 ***	0.04 ***	0.01 ^NS^	0.01 ^NS^	0.02 *	0.05 ***
M × I × D	0.04 ^NS^	0.06 ***	0.03 *	0.03 ^NS^	0.04 *	0.05 **	0.04 *	0.10 ***

Results were generated using 100,000 iterations, for the experimental mow height treatments (denoted as Mowing or M), water supplied (denoted as Irrigation or I) and date of measurement (denoted as Date or D), with interactions with VQ, NDVI, and image metrics are presented. The treatments included four mowing heights (2.5, 5.0, 7.5, and 10.0 cm), three water application levels [(100 × ET_a_ (actual evapotranspiration), 65 × ET_a_, and 30 × ET_a_)], and eight weekly data collections. The file format and image correction applied are noted above the variable name. ηp2 values measure percentage of variance accounted by each treatment effect where higher values indicate more significant effect sizes, respective values above 0.01, 0.06, and 0.14 indicate small, medium, and large. Significant differences for means are indicated as not significant, *p* ≤ 0.05, *p* ≤ 0.01, and *p* ≤ 0.001 using the symbols NS, *, **, and ***, respectively.

**Table 5:** Coefficient of variation (CV) for replicate images across file format and image corrections.

	%Y	%G	DGCI	HSVi	BA	COMB2
JPG	0.0154	0.0084	0.0033	0.0032	−0.0059	0.0131
JPG CC	0.0131 ^NS^	0.0058 *	0.0039 ^NS^	0.0042 *	−0.0066 ^NS^	0.0206 ^NS^
JPG LC	0.0162 ^NS^	0.0085 ^NS^	0.0060 ***	0.0042 ^NS^	**−0.0063** ^NS^	0.0184 ^NS^
JPG LC CC	**0.0141** ^NS^	0.0059 *	0.0057 ***	0.0051 ***	−0.0070 ^NS^	0.0326 *
TIF	0.0162 ^NS^	**0.0081** ^NS^	0.0033 ^NS^	0.0031 ^NS^	−0.0059 ^NS^	0.0127 ^NS^
TIF CC	0.0133 ^NS^	0.0053 **	**0.0060** ^NS^	**0.0039** ^NS^	−0.0065 ^NS^	0.0195 ^NS^
TIF LC	0.0154 ^NS^	0.0080 ^NS^	0.0054 ***	0.0036 ^NS^	−0.0063 ^NS^	0.0143 ^NS^
TIF LC CC	0.0129 ^NS^	0.0053 ***	0.0063 ***	0.0048 ***	−0.0070 ^NS^	**0.0354** ^NS^
Average	0.0236 ^NS^	0.0118 ***	0.0057 ***	0.0052 ***	−0.0088 ^NS^	0.0142 **

Bold values note the image correction that resulted in the highest correlation between the image-based calculation variable and its associated reference (Table 2). The median CV values for selected calculated metrics across each image correction were tested for significant difference using a one-way Chi-square approximation of the non-parametric Kruskal–Wallis test of medians and Dunn’s joint ranks test with Bonferroni adjustment and the JPEG file format as control. Significant difference in median value is noted as not significant, *p* ≤ 0.05, *p* ≤ 0.01, and *p* ≤ 0.001 using the symbols NS, *, **, and ***, respectively.

## Data Availability

Datasets are supplied in the Supplementary Materials and at https://agdatacommons.nal.usda.gov/articles/dataset/Data_from_Visualizing_Plant_Responses_Novel_Insights_Possible_through_Affordable_Imaging_Techniques_in_the_Greenhouse/26527447, accessed on 13 August 2024; images are available upon request.

## Supplementary Materials

The following supporting information can be downloaded at: https://www.mdpi.com/article/10.3390/s24206676/s1. Supplementary S1: F-values. Supplementary S2: *p*-values for experimental effects ANOVA (Table 4), nine additional individual time series charts. Supplementary S3: of BA, %C, %G, DGCI, HSVi, NDRE, NIR, RED, and RE metrics. Supplementary S4: Additional discussion text. Supplementary S5: Raw data.

## Appendix A. Description of Environmental Measurements

Using a simple methodological approach that involved common inexpensive products, 15.2 × 30.5 cm lysimeter cylindrical containers were assembled from schedule-40 polyvinylchloride pipe and endcaps, then filled with a 10:90 peat-to-sand ratio with USGA (https://www.usga.org/, accessed on 4 July 2023)-specification sand (sandy loam) as a growth medium. Plugs from TifTuf Bermuda sod were planted in each container and allowed to establish over a 7-week period. Irrigation and mowing treatments were applied weekly during the experiment period. Greenhouse conditions were managed by a Wadsworth Control Systems (Arvada, CO, USA) aspirated temperature controller setting of 33 or 26 °C (day or night, respectively). Micro-meteorological conditions were recorded using Campbell Scientific (Campbell Scientific Corporation, Logan, UT, USA) data loggers (CR3000 and CR1000x, respectively) in each greenhouse and included air temperature and relative humidity measured by the HC2S3 Rotronic sensor (Rotronic Instrument Corp, Hauppauge, NY, USA) nested in an RM Young ten plate solar radiation shield (R.M. Young Comp., Traverse City, MI, USA). Full-spectrum solar radiation was measured by the Apogee SP-110 pyranometer, and photosynthetically active radiation was measured with the Apogee SQ-500 quantum sensor (Apogee Instruments, Inc., Logan, UT, USA). Starting at trial initiation, plants were grown in two adjacent greenhouses over eight-week periods and were exposed to 13.4 and 14.4 °C average daily growing degree days (GDDs, using a base of 10 °C) for a total of 757 and 794 °C GDDs, respectively. Regarding solar energy, the plants experienced a 150-unit daily average for 8268 total µmols m^−2^ s^−1^ photosynthetically active quantum flux radiation (PAR). However, across the experiment period the average daily temperature reduced from 26 to 21 and 28 to 24 °C in the two respective greenhouses, and the PAR reduced from a daily average of 194 to 137 µmols m^−2^ s^−1^, signifying the autumn seasonal environment.

**Table A1:** Average and cumulative environmental variables that contextualize the growing conditions.

	Day	Night	GDD	GDD Total	PAR	PAR Total	VPD
	C	C	C	C	µmols m^−2^ s^−1^	µmols m^−2^ s^−1^	kPa
Greenhouse 1	28.6	20.3	13.4	757	147	8080	2.07
Greenhouse 2	29.6	20.6	14.4	794	154	8456	2.16
