# Supplementary material for: Visualizing Plant Responses: Novel Insights Possible Through Affordable Imaging Techniques in the Greenhouse
Source: Sensors (Basel). 2024 Oct 17;24(20):6676. doi: 10.3390/s24206676 (PMC11511021; doi:10.3390/s24206676)
Supplement: Supplementary file 1 [file sensors-24-06676-s001.zip › Supplementary S4.pdf]

Supplemental Discussion text:

Visualizing Plant Responses: Novel Insights Possible through Affordable Imaging Techniques in the Greenhouse

Dynamic selection is an easy way to determine and verify color-based cover classification areas. These areas can be parametrically refined using gradient-based selection masks, included in TurfAnalyzer 1.0.4 software and common in commercial image editing applications like Adobe Photoshop. By manipulating the criterion control, users can update a selection on the display of an evaluation image and visually assess whether the proper regions of interest are captured. This dynamic process enables users to determine if a particular image metric is responding to an intended color or feature while ensuring that unwanted colors and features are excluded. A similar computational process can be developed with Python 3 scripts and was used for this paper. Given the variability in illumination conditions and camera-specific factors such as sensor characteristics and color processing algorithms, it is recommended to perform iterative visual assessment and adjustment of segmentation parameters to support accurate identification and selection of target chromatic values in the image data. This process can certify that the correct regions are selected based on the predefined parameter set. Related, the export of a validation images, which consist of the intersection between the original image pixels and the selection mask (including its inversion) enables a zoomed-in view for more detailed inspection of feature transition edges. Detailed scrutiny, to the limit of the pixel resolution, can reveal whether the target feature area has been fully segmented by the classification parameters employed. TurfAnalyzer 1.0.4 software outputs image files that display mask intersection results. However, these images are of reduced resolution and do not depict the original pixels themselves. The Python approach used in this paper enabled the extraction of full-resolution pixel selections output to individual image files that could be closely evaluated.

Though the simple image-based approach in this paper used to identify basic cover and color features was successful, limitations persist, and several improvements can be made. First, to enhance color accuracy and consistency in subsequent investigations, the illumination source will be upgraded to a professional-grade LED option with a high color rendering index [147,148], such as exemplified in the Waveform Lighting Absolute Series ™ product line. Second, considering that the camera selected was released in 2013, a more modern camera may incorporate improved technology and increase the quality of the base data. Third, although no errors were detected, adjusting the camera lens aperture 3.5 F-Stop to a higher setting of around f/8 to f/11 would elongate focal depth of field, decrease lens vignette artifacts, optimize lens sharpness, and reduce chromatic aberration without inducing major diffraction. Fourth, improved computational pipelines capable of handling RAW-based imagery [149] are anticipated to become more widely available and easier to use in the future.

While uncompressed TIFF format imagery was processed in the Python pipeline used in this study, larger files are more challenging to manage, and there is less software support for uncompressed file formats compared to the common JPEG form. Yet handling all the image information generated by the camera exposure is expected to improve data quality. Likewise, advancements in color correction [150–152] software are expected to become more accessible over time. The authors plan to obtain a license and use the Adobe Lightroom commercial software (and they may use the Python color-checker-detection library) to make color corrections in future experiments. A more robust color correction procedure could enhance data quality. Given the high image resolution capabilities of modern cameras, JEPG compression of high-resolution imagery may be acceptable for proximal measurement [153–156]. Since camera and lighting limitations are expected to introduce some level of error [157], a careful assessment is necessary to determine whether inherent camera-induced representational errors are acceptable. The value of corrections should be weighed against their implementation costs and the potential introduction of new error sources. Refinement of the corrections methodology is essential. This study used a sensitivity approach to compare the effects of eight individual image types on six image metric correlations with eight reference measurements, including VQ and NDVI. The study indicates that %Y, BA, and HSVi have the potential to differentiate the effects of experimental treatments in addition to the more traditional %G, DGCI and COMB2. However, it also highlights challenges in quantifying the effects of image corrections across diverse metrics.

The effectiveness of color corrections and other image adjustments to enhance data quality for a specific plant phenotyping collection is ambiguous. While corrections are sometimes made, evidence of improvement may be lacking [158,159]. There are well-developed theoretical best practices for corrections [160–165], but additional guidance could be provided to quantify the effects of image data adjustments under various real-world conditions. This would help understanding and determination of which imagery correction procedures are necessary in a particular circumstance. For instance, Chopin demonstrated that their camera profile for proximal field imagery was better fitted using a least squares quadratic transformation rather than linear [166]. Despite the challenges posed by a diverse range of camera products, application spaces, and vegetative variability, clear standards for operations specific to various applications would be of benefit to users. The provision of clearer guidelines and empirical evidence to describe the effects of image adjustments would allow practitioners to make more informed decisions, ultimately enhancing the quality and reliability of plant phenotyping data.

Consider that a RAW file format allows post-processing of the total camera-generated data. Yet a lossless TIFF file format will retain all the image pixel color values. On the other hand, while the JPEG format is lossy, high-resolution imagery could mitigate the effects of compression loss and may therefore be acceptable for plant phenotyping functions. Although image data corrections are important to consider [167], they can be

challenging to implement, and their effects could be difficult to quantify and report.

As part of the exploration process, consideration was given to the Python-based plant phenotyping research software PlantCV 4.5 (https://plantcv.danforthcenter.org/, accessed 7 November 2023). Despite its provisions for color correction and many analysis functions, its design did not align with the generic approach of this investigation. Traditional PlantCV operation requires a specific color correction panel to be present in each image, which can pose a geometric obstacle inside a compact lightbox. Although having a dedicated correction reference panel in each image provides for superior batch correction compared to using only one reference image for an entire set, the production and processing of dedicated correction panel views increases complexity, reduces the usable image analysis view space, and may not overcome errors due to inconsistent illumination across a single image. Professional software, such as Adobe Photoshop and Lightroom, are often used to color-correct imagery [168–171]. However, these tools were not utilized in this study absent their commercial license. Therefore, although color correction is advantageous for comparing imagery taken at different times, in different illumination scenarios, and from different cameras, the benefit of color correction must be weighed against the additional complexity it introduces. Moreover, there is a possibility that color correction can introduce errors that may be difficult to quantify. Thus, the decision to implement color correction should be made judiciously, considering its potential positive impact and the trade-offs involved.

Achieving geometric regularity in images through lens distortion correction is a common enhancement, but it may be challenging to implement. An improved lens correction process could aid higher-quality data results in plant phenotyping [172]. Although the Lensfun database 0.3.3 of lens artifacts (https://lensfun.github.io/, assessed 4 April 2024) was available for the Nikon lens used, authors found the correction to be insufficient even after a second manual adjustment was included. This may be due to minor manufacturing imperfections unique to the specific lens used. While RawTherapee 5.9 (https://rawtherapee.com/, assessed 28 March 2023), GIMP 2.10.36 (Crunchbase Company, Charlotte, NC, USA), and Pablo d' Angelo's Hugin software Hugin - Panorama photo stitcher 2023.0.0 (https://hugin.sourceforge.io/, assessed 10 April 2024) were tested for correcting lens distortion, the Python Discorpy package 1.6.0 was selected as the most effective solution and offered well-documented tutorials. However, further refinements may still be possible because LC with Discorpy brightened as well as geometrically corrected images (Table S3).

Table S3. Average effects of LC on HSV and RGB channel values.

Hue	Saturation	Value
0.0%	-3.8%	11.6%

Red	Green	Blue
12.2%	11.6%	14.7%

[6] Nominal changes were observed for Hue angles (0.002%) between JPG and JPG LC, while more substantial alterations occurred with a decreased Saturation and increased brightness Value. This equated to an increase in all RGB values. Consequently, the accuracy of the corrections, a potential new source of error, the added challenge in making corrections for each camera setup, and a subsequent benefit from LC must be balanced together. A straightforward LC result verification is needed to better understand the extent to which LC may improve or degrade a particular imagery set.

A lightbox with many LEDs can provide consistent illumination to facilitate the capture of extensive gridded optical data [173–175], but its usage imposes major limitations on sample throughput. Inserting each individual lysimeter into the lightbox for imaging was considerably slower (approximately 60 seconds) than the VQ assessment (approximately 3 seconds) or NDVI measurement (approximately 15 seconds). Although a robotic high-throughput plant phenotyping greenhouse can address this restriction [176–181], its adoption comes at substantial monetary and complexity cost, albeit more generic versions are being developed [182]. Some experiments have imaged multiple greenhouse plants outside the lightbox omitting robotics [95,158] but the ambient illumination is likely more varied across each image. Even when a color correction panel is present within each image collected, inconsistent lighting across the entire image can compromise the accuracy of color correction. Hence, improved methods may entail consistent illumination [183] and enable plants to be imaged without the need for relocation.

Micrometeorological measurements of the growing environment are important in plant phenotyping. At a minimum, measurement of photosynthetic radiation, along with sensible and latent heat, provides a context for plant growth (Appendix A). Even minor discrepancies between the expected environmental values, such as air temperature, and the actual values measured at the precise location of the plants, can accumulate over a growing season and impact results. There can also be short-duration events, such as loss of greenhouse control or abrupt outside weather changes, which can affect plant growth inside a greenhouse. These events may go unnoticed without continuous environmental measurements at sufficient temporal resolution. In this study, greenhouse environments were measured at a frequency of 1Hz, ensuring a comprehensive characterization of the growing conditions. However, the plant images were only captured every two weeks. Increasing the frequency of image capture would facilitate the association of temporal growth indicators present in the imagery data with the measured environment. This would lead to better understanding of environmental effects on observed biological results.

Optical cameras only measure in the RGB color spectrum (approximately 420 to 700 nm). Beyond this range lies spectral information that is significant for researchers but surpasses the detection capacity of standard cameras. Consequently, the optical camera alone cannot detect the complete spectral reflection of vegetation. Another limitation of the camera sensor is the 2-D representation it generates. Although photogrammetric or structure-from-motion techniques can be used to amalgamate multiple images into 3-D point clouds, incorporating a second view perpendicular to the nadir would provide more plant information and allow for a basic 2.5-D model with just two images, and reduced compute requirement.

Incorporating an up-looking optical diffuser with filters that transmit the RGB frequencies of the camera detector, similar to the design of some UAS multi-spectral cameras [184] like the MicaSense Altum or RedEdge cameras (AgEagle Aerial Systems Inc., Wichita, KS, USA), which are designed specific to their spectral sensitivities, would enable a solar cosine correction [185] frame by frame onboard the camera. This may diminish the value of color correction post-processing and greatly expedite the information process while increasing data quality.

The NDVI measurement used in this paper was derived from a unique active optical sensor technology. Unlike passive sensors, this technology allows measurements in various lighting conditions. Although the instrument used provides a set of raw values and basic statistics from its high-frequency sampling, it only offers a single data point per measurement. An NDVI camera would provide additional spatial information through its pixel grid; however, considerations related to ambient light would come into play. The authors are unaware of any active NDVI camera product.
