# Supplementary material for: Visualizing Plant Responses: Novel Insights Possible Through Affordable Imaging Techniques in the Greenhouse
Source: Sensors (Basel). 2024 Oct 17;24(20):6676. doi: 10.3390/s24206676 (PMC11511021; doi:10.3390/s24206676)
Supplement: Supplementary file 1 [file sensors-24-06676-s001.zip › Supplementary S5/ReadMe.pdf]

Description of data for the paper “Visualizing Plant Responses: Novel Insights Possible through Affordable Imaging Techniques in the Greenhouse”.

The file “Data_.csv” contains experimental treatment classifications and measurements, as well as reflectance data and imagery calculations.

1. Date - the day on which the imagery data and associated variables were collected
2. Height – the height in cm of the turf grass mowing treatment
3. IRR – the percentage of replacement water provided
4. Rep – the replication number
5. Run – the 72 lysimeters were halved and separated into two adjacent greenhouses whereby the whole experiment was duplicated. Run number is one or two for the two parts.
6. VQ – human assessed visual quality rating
7. Treatment – a combined term of the water supplied and the mowing height
8. mg/day – the weight of clipping biomass removed due to the mowing treatment normalized by day
9. mm/day – the volume of 100% water replacement provided
10. Collection – the imagery collection starting with collection 2 (C2), The run number part (P1 or 2), and the week of the eight-week experiment starting with week 1 (W1).
11. NDRE – Normalized difference in Red-Edge vegetation index data
12. NDVI – Normalized difference vegetation index data
13. RE – Red-Edge reflectance data
14. NIR – Near Infrared reflectance data
15. R – Red reflectance data
16. TIME – the time reflectance data was measured
17. N – the number of samples taken by the active reflectance sensor
18. MAXNDRE – the maximum NDRE measured
19. MAXNDVI – the maximum NDVI measured
20. MINNDRE – the minimum NDRE measured
21. MINNDVI – the minimum NDVI measured
22. STDNDRE – the standard deviation of the NDRE measurement
23. STDNDVI - the standard deviation of the NDVI measurement
24. CVNDRE – the coefficient of determination of the NDRE measurement
25. CVNDVI – the coefficient of determination of the NDVI measurement
26. Week – the week of the experiment measurement period
27. LYS – the number of the lysimeter which is the lysimeter tracking name
28. Sample – the order in which images were processed

Image data is presented for several metrics, including two file types and six corrections.

1. JPG – the standard JPEG file compression
2. TIF – the standard TIFF in lossless conversion from NEF RAW file type
3. LC – geometric Lens Correction
4. CC – Color Correction
5. LC_CC – the combination of Color Correction and Lens Correction

Several calculations were made on the imagery

1. BA – the LABCIE b* to a* ratio
2. COMB2 – the combination vegetation index #2 taken from *S. Singh et al., "Genetic Variability of Traffic Tolerance and Surface Playability of Bermudagrass (Cynodon spp.) under Fall Simulated Traffic Stress," horts, vol. 59, no. 1, pp. 73–83, 2024, doi: 10.21273/HORTSCI17488-23*
3. DGCI – the dark green color index from *Rorie, R. L., Purcell, L. C., Karcher, D. E., & King, C. A. (2011). The assessment of leaf nitrogen in corn from digital images. Crop Science, 51(5), 2174-2180*
4. %G – Is the fraction of green area cover as determined by the HSV imagery values in the paper
5. %Y – Is the fraction of yellow area cover as determined by the HSV imagery values in the paper
6. %C – Is the fraction of living plant material as determined by the HSV imagery values in the paper

The file "AVG_.csv" contains a list of the image calculations and image file format and corrections. The data in this file is also contained in the "Data_.csv" file, however the "AVG_.csv" file has been reformatted to facilitate an analysis of the effects of the file format and corrections on the mean values of the calculations.

1. Type – is the file type and correction applied

The file "CV_.csv" contains the coefficient of variation for calculated image values organized by file type and correction applied. This data was used to test how file type and corrections affected the variation in sets of three replicate images.

1. Type – is the file type and correction applied
2. Run – is the weekly collection and experiment part string designation
3. The image metrics are the same as in "Data_.csv"
