# Supplementary figures and images for: Visualizing Plant Responses: Novel Insights Possible Through Affordable Imaging Techniques in the Greenhouse
Source: Sensors (Basel). 2024 Oct 17;24(20):6676. doi: 10.3390/s24206676 (PMC11511021; doi:10.3390/s24206676)

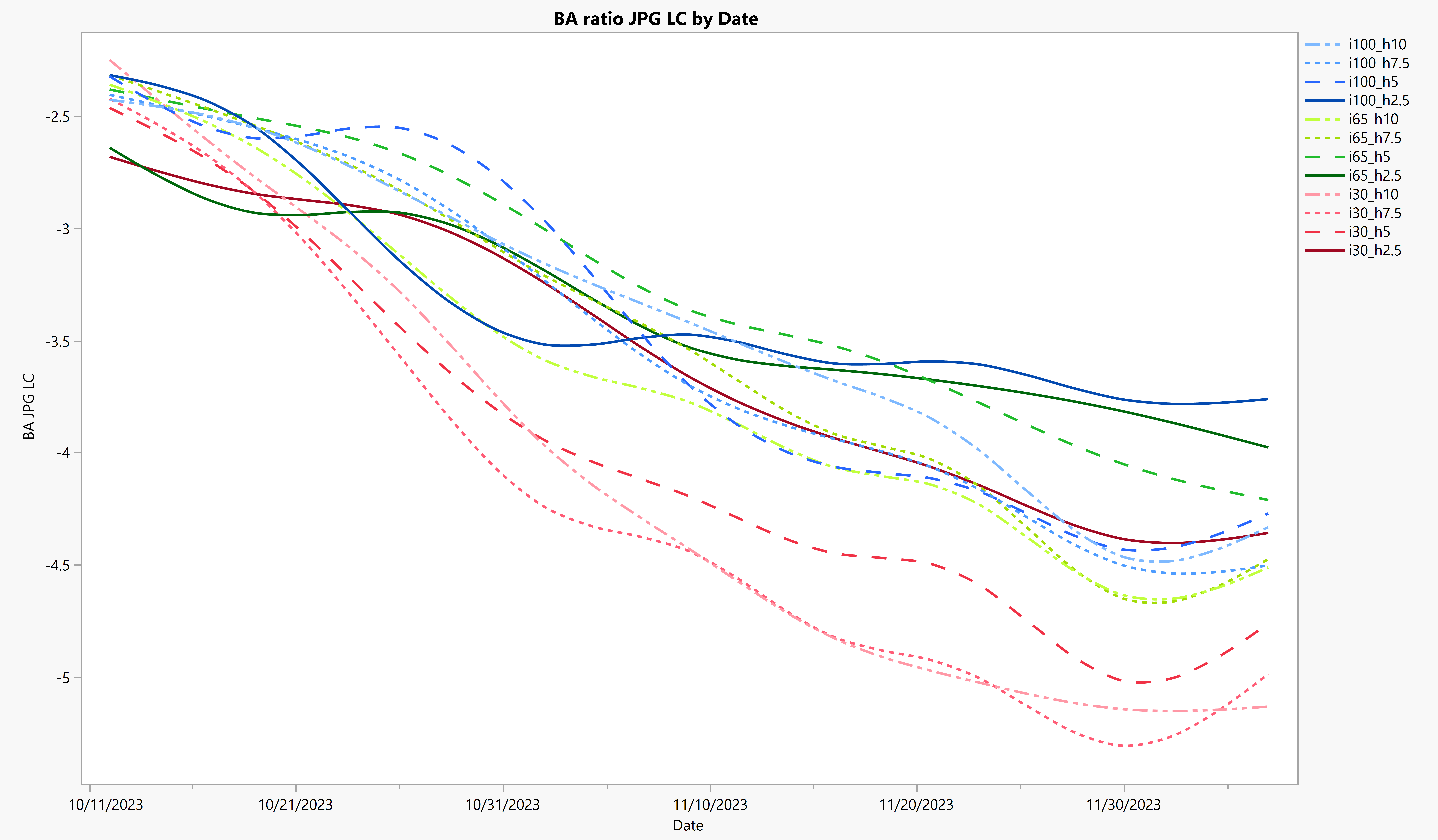

Supplement: Supplementary file 1 [file sensors-24-06676-s001.zip › Supplementary S3/BA.png]

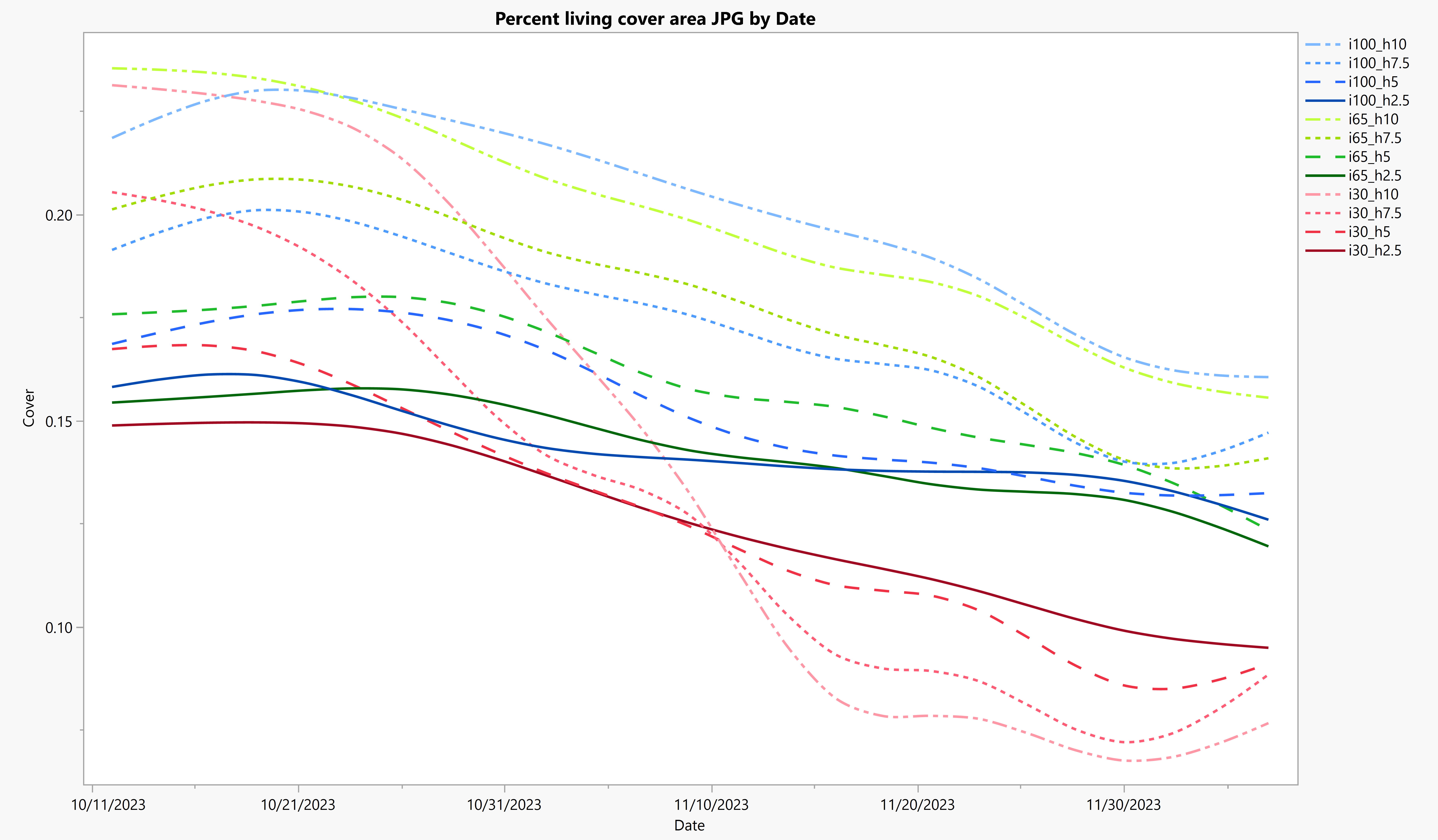

Supplement: Supplementary file 1 [file sensors-24-06676-s001.zip › Supplementary S3/Cover.png]

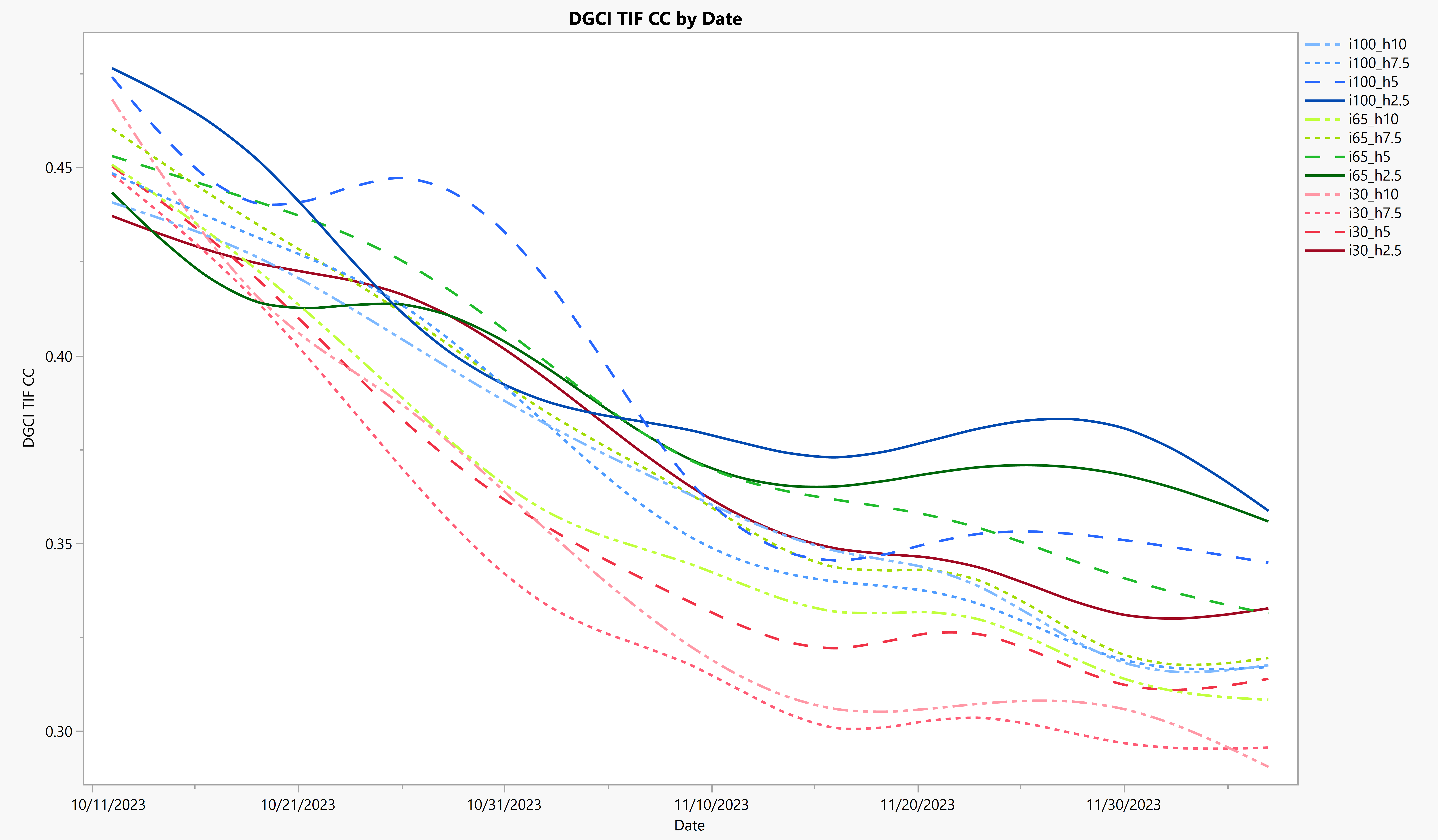

Supplement: Supplementary file 1 [file sensors-24-06676-s001.zip › Supplementary S3/DGCI.png]

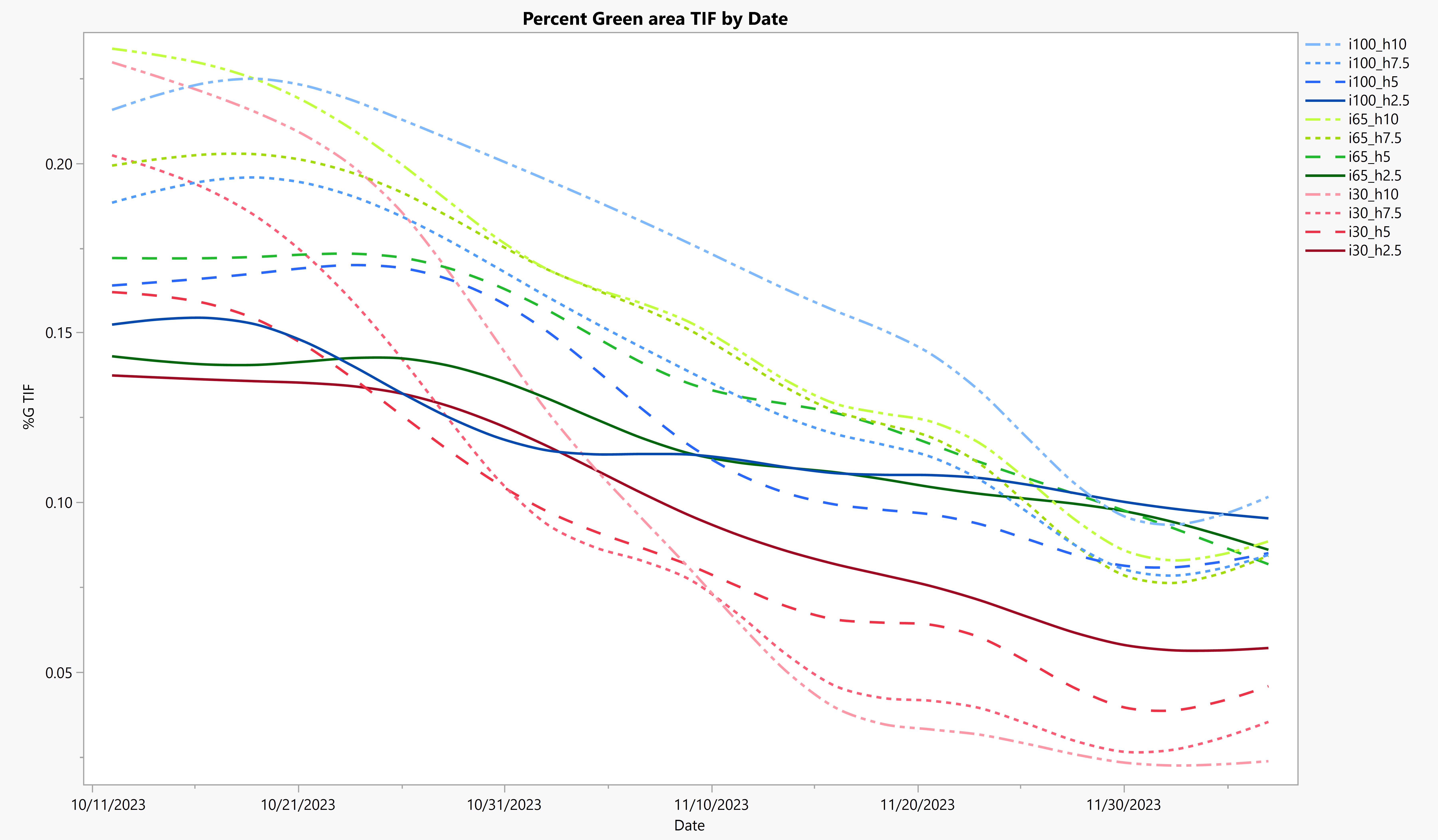

Supplement: Supplementary file 1 [file sensors-24-06676-s001.zip › Supplementary S3/Green.png]

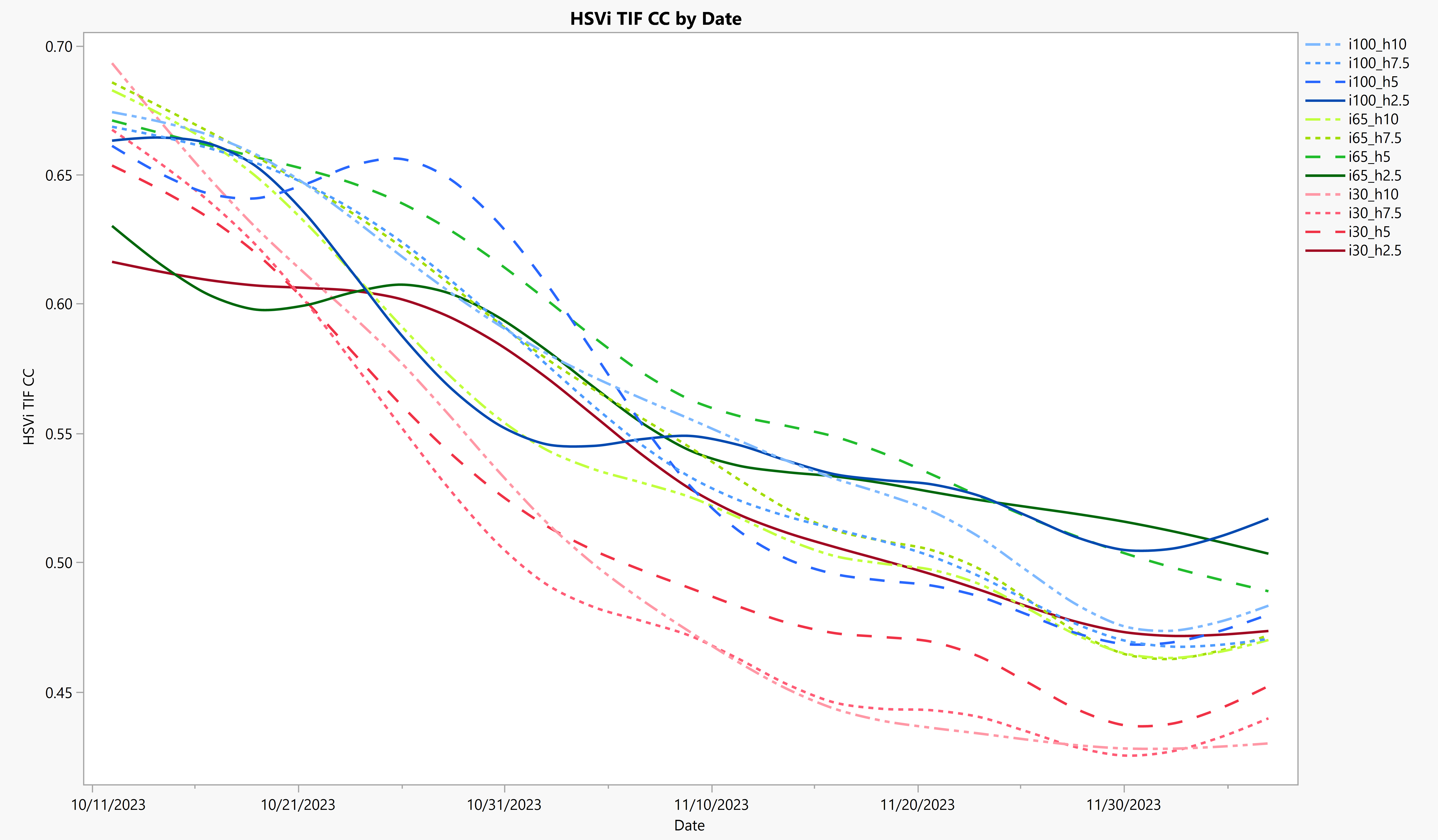

Supplement: Supplementary file 1 [file sensors-24-06676-s001.zip › Supplementary S3/HSVi.png]

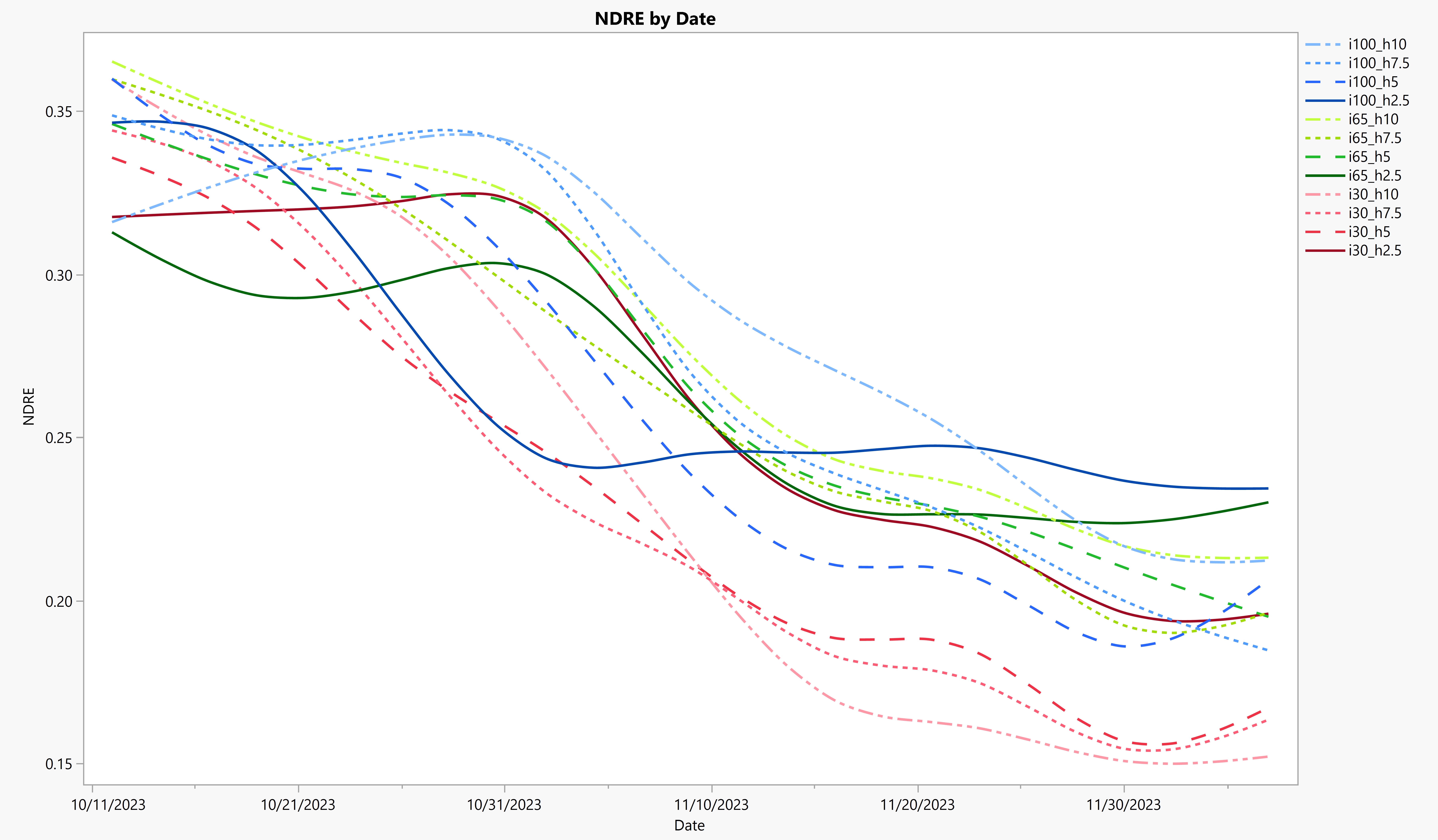

Supplement: Supplementary file 1 [file sensors-24-06676-s001.zip › Supplementary S3/NDRE.png]

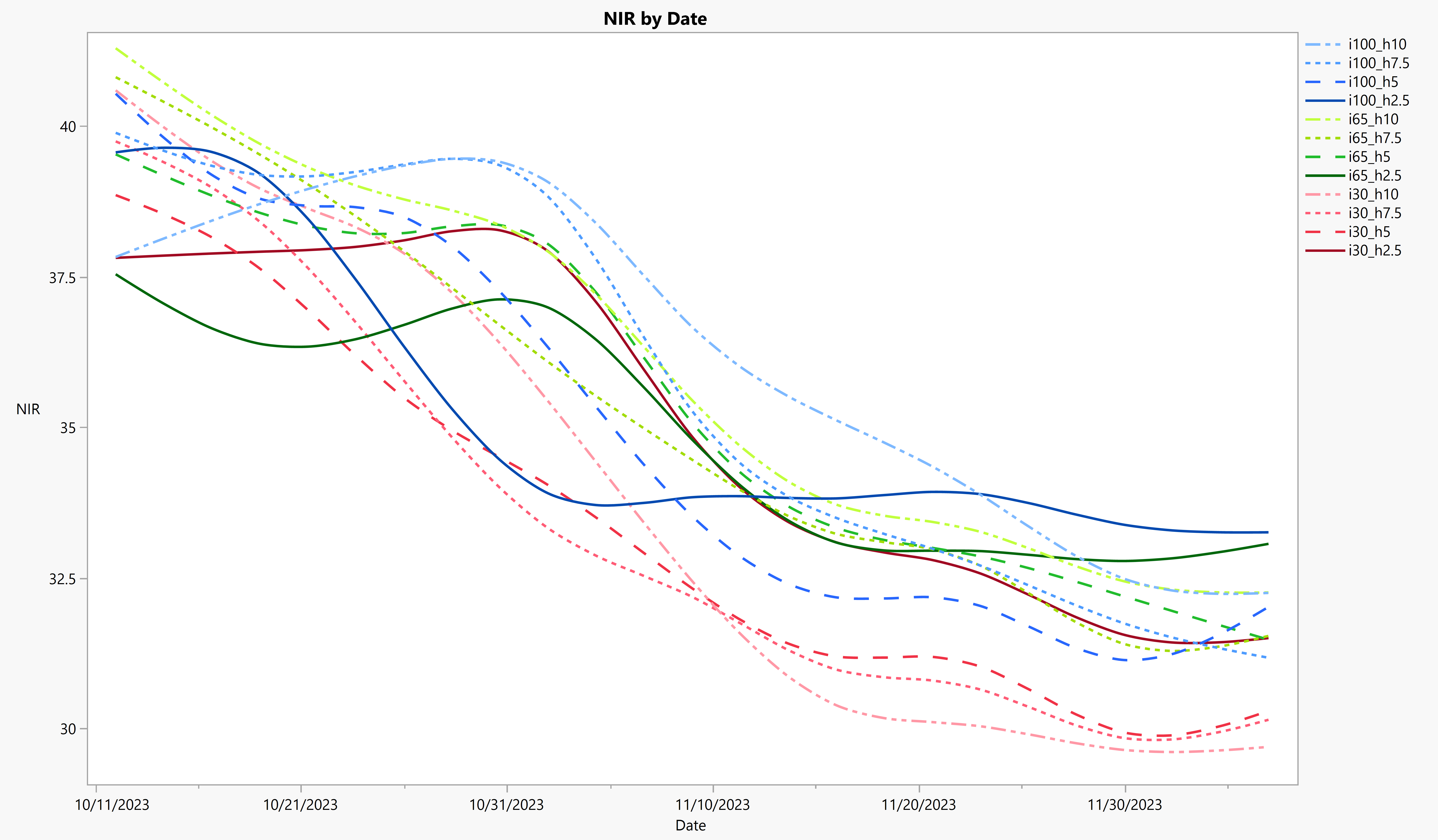

Supplement: Supplementary file 1 [file sensors-24-06676-s001.zip › Supplementary S3/NIR.png]

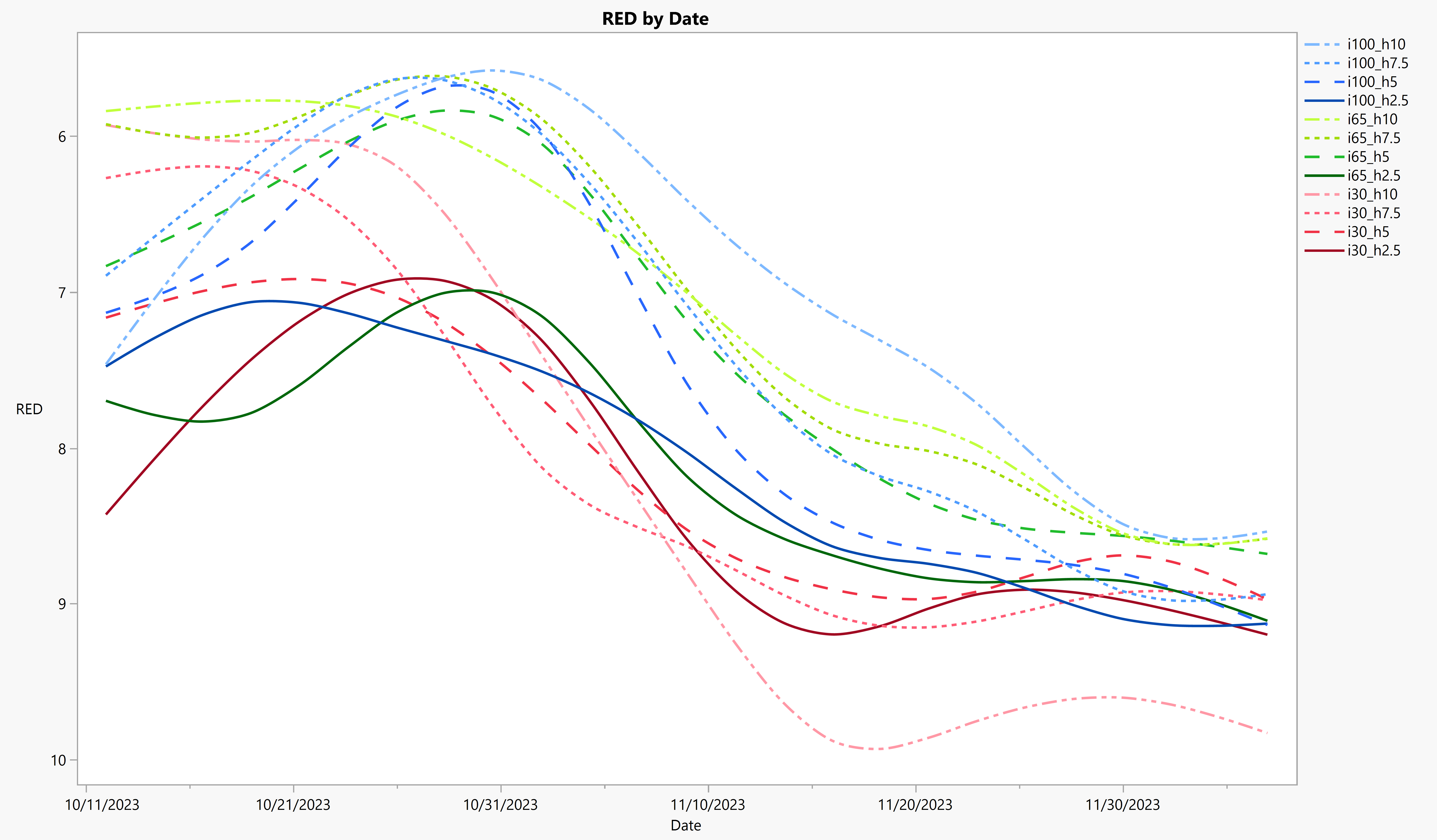

Supplement: Supplementary file 1 [file sensors-24-06676-s001.zip › Supplementary S3/RED.png]

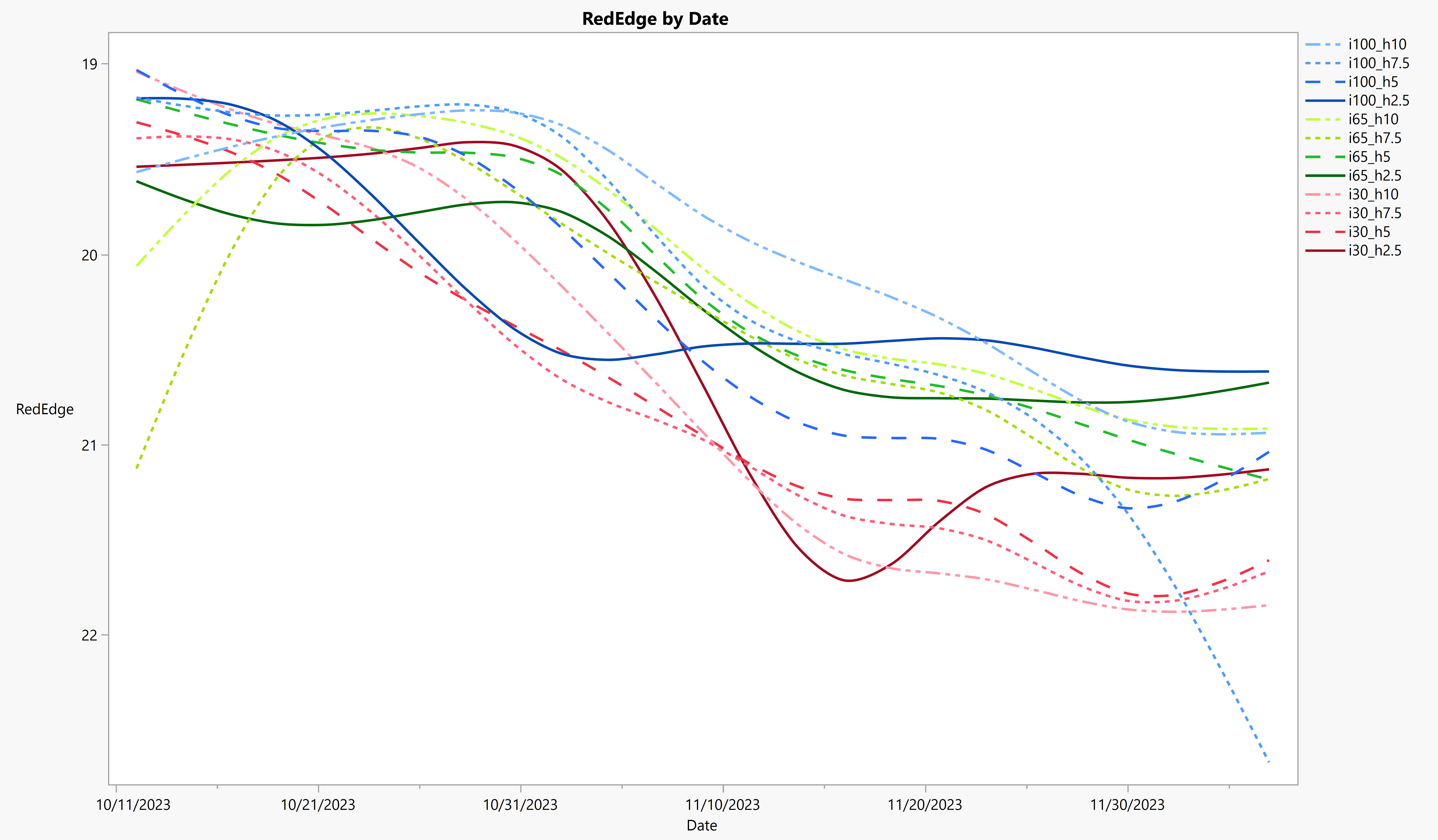

Supplement: Supplementary file 1 [file sensors-24-06676-s001.zip › Supplementary S3/RedEdge.png]
